# Aula Verde (tree room) as a link between art and science to raise public awareness of nature-based solutions

**DOI:** 10.1038/s41598-024-51611-9

**Published:** 2024-02-06

**Authors:** A. Conte, R. Pace, Q. Li, S. Carloni, A. Boetzkes, L. Passatore

**Affiliations:** 1 Climate Art Project, 00185 Rome, Italy; 2 Futurecologies Startup, 00167 Rome, Italy; 3 Institute of Meteorology and Climate Research, Atmospheric Environmental Research (IMK-IFU), Karlsruhe Institute of Technology (KIT), 82467 Garmisch-Partenkirchen, Germany; 4 EURAC Research, Institute for Renewable Energy, 39100 Bolzano, Italy; 5 Department of Rehabilitation Medicine, Nippon Medical School, Tokyo, 113-8603 Japan; 6 The Japanese Society of Forest Medicine, Tokyo, 113-8603 Japan; 7 Research Institute on Terrestrial Ecosystems (IRET), National Research Council (CNR), Area della Ricerca CNR Roma 1, Via Salaria km 29.300, 00015 Monterotondo, Rome, Italy; 8 University of Guelph, Guelph, ON N1G 2W1 Canada

**Keywords:** Climate-change adaptation, Psychology and behaviour, Sustainability

## Abstract

Nature-based solutions inherently require a multifaceted perspective that encompasses diverse fields. The aim of this project is to develop more effective nature-based solutions, climate action and environmental awareness by breaking down boundaries between disciplines and fostering a co-creative process. Concepts of ecology and urban forestry were combined with the research on political ecology, environmental humanities, land art, regenerative art, performing art, participatory art, and more-than-human art. This process resulted in the creation of Aula Verde Aniene. It is located in an urban park in Rome and consists of a stand of trees arranged in circles with a specific design to give the perception of being in an outdoor vegetated room. The project activities involved community participation through art performances and citizen science initiatives. Regulating and cultural ecosystem services of Aula Verde were assessed using i-Tree Eco software and citizens’ surveys. Beyond numerical descriptions of ecosystem services, the manuscript introduces shinrin-yoku as a practice to raise awareness of nature. The distinctive approach here described contributed to convey a sense of belonging to the ecosystem to citizens. The project framework and study findings have been developed to formulate policy recommendations and disseminate a format that can be adapted to diverse locations.

## Introduction

Over recent decades, different research domains have focused on the use of green resources to address liveability, sustainability, climate change mitigation and adaptation in urban areas.

Since 2000, we have officially become an urban species. The urban population worldwide grew from just 746 million in 1950 to 3.9 billion in 2014, according to the United Nations Population Division. By 2050, 75% of the world’s projected 9 billion population will live in cities^1,2^. Urban areas around the world are increasingly investing in networks of urban forests, green roofs, gardens, and other forms of nature-based solutions for their benefits^3^. Meanwhile, there has been wide research on the use of natural components and their multiple functions to address liveability, sustainability, and climate change mitigation and adaptation in urban areas. The concepts of urban forests, ecosystem services (ESs), green infrastructures and nature-based solutions (NBSs) have populated the scientific literature in recent decades, evolving, diversifying, and gaining popularity depending on the period and country^4^.

Within the present manuscript, we will focus on the concept of NBSs, which is one of the most recent and widespread paradigms indicating “actions to protect, sustainably manage and restore natural and modified ecosystems in ways that address societal challenges effectively and adaptively, to provide both human well-being and biodiversity benefits” as defined by the International Union for Conservation of Nature^5^.

**Figure 1:**
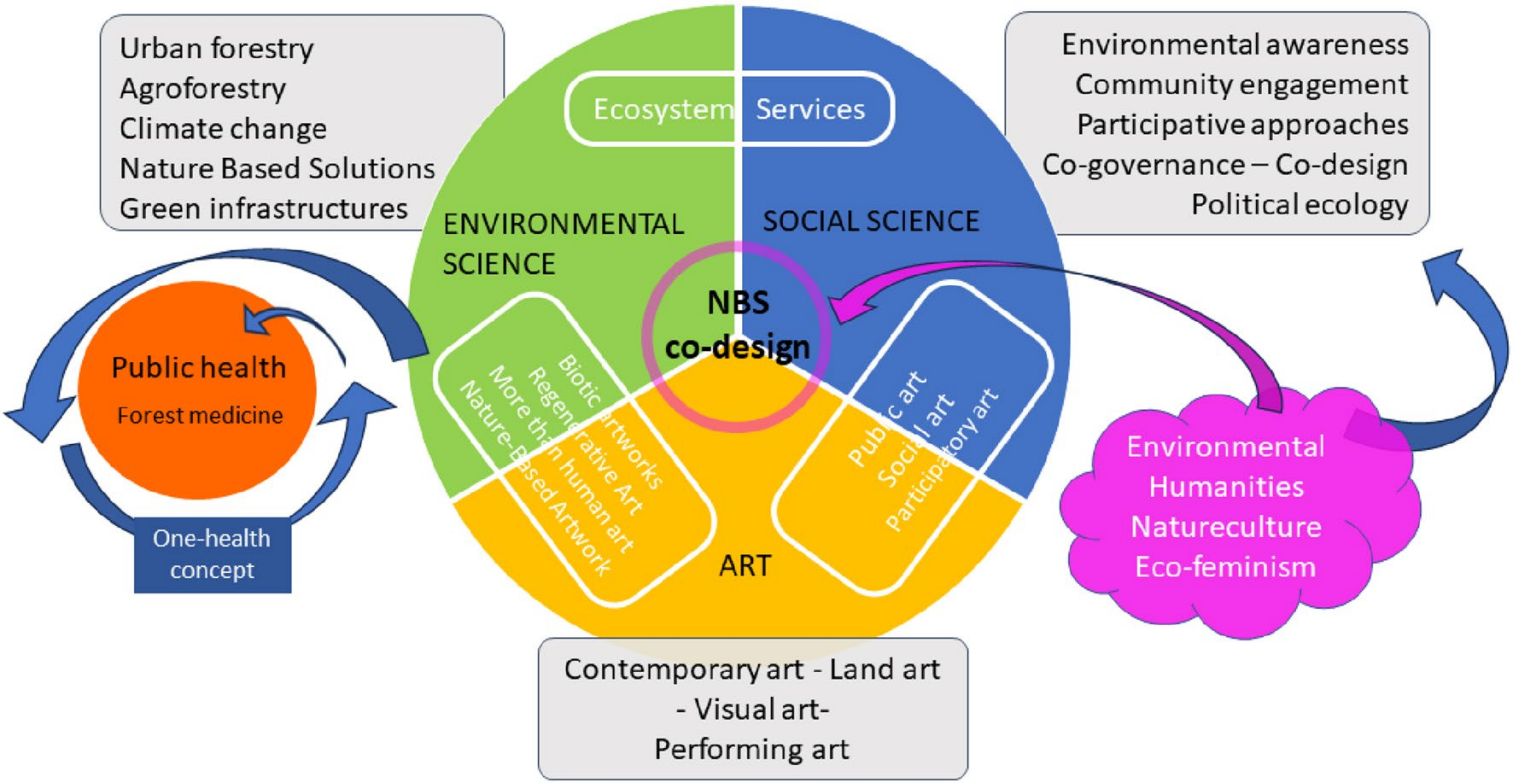
Theoretical framework of the manuscript. The diagram shows the collaboration between different disciplines and the key concepts related to the work. It describes an innovative version of NBS—co design process that take in consideration: art practices, social sciences, political ecology, one health theories and environmental science.

The theoretical framework of the paper, as depicted in Fig. 1, is also influenced by contemporary philosophy, political ecology and ecofeminist theories. The manuscript is based on assuming the end of the old dichotomy between nature and society and considers a complex collective of human and non-human entities^6^, “other-than-human critters”^7^ and “more-than-human” geographies^8^. These ideas align with the "one-health" holistic approach that is tightly linked to the epidemiology competency framework. This concept encourages and advocates for the interdependence, coexistence, and evolution of living beings and their environment, considering the health of mankind, animals, and ecosystems as an indissoluble whole^9^. The dichotomy between the concepts of nature and culture in the Western world has been instrumental to the objectification and exploitation of natural resources and colonisation of the global south^10^. Humans are nature taking conscious of themself^11,12^. The Nature/Culture approach^13^ or the natureculture perspective^14^ are more helpful in conceiving interrelationships between different forms of living beings and ecosystems. Considering the complexity of the interrelation within the ecosystem components, it is crucial to develop a post-anthropocentric view^15^ or better a more symbiotic and ecocentric approach^16^ based on kinships^17^ and an interspecies mutualism^18^. The project has been greatly impacted by philosophical theories from its conception. This includes the application of frameworks for ecological and regenerative art, as well as for green infrastructure and nature-based solutions.

The actual framework for nature-based solutions implementation strongly requires a transdisciplinary collaboration. To effectively address the challenges in implementing NBS, it is recommended the adoption of a learning-by-doing approach based on collaborative efforts across different disciplines^19^. This implies that scientists may need to undertake a new role, including developing frameworks and methods that facilitate cooperation and co-creation across diverse sectors, disciplines, and social practices^20^. Recent articles explored the incorporation of co-creation and participatory processes, along with the infusion of social and cultural activities, into the framework of NBS design to enhance their overall effectiveness. Nevertheless, these processes did not explore the potential integration of contemporary art practices. The use of artistic language fosters a more emotional interpretation of NBS, leading to the development of fresh perspectives in people’s minds and an enhanced connection with the other species and non-human entities. In this manuscript, we have explored the “Aula Verde” concept as a means to convey the scientific message of NBS and promote the processes of environmental co-creation and education. Our approach not only promotes ecosystem and cultural services but also serves to strengthen the reconnection between humans and non-humans through art practices.

This connection is essential in shifting from an anthropocentric to an ecocentric perspective, which is a crucial measure to curb the anthropic extractivist approach that causes most of the environmental challenges of our time.

The project described in this manuscript, which merges science and art, offers a unique way of communicating to citizens a sense of belonging to the ecosystem. This should foster the will of taking care of the common spaces and promoting the interspecies mutual aid which constitutes the foundation of political ecology.

The aimed ecological turn will not happen only as consequences of technical innovations but also through eco-social and cultural changes.

The manuscript, influenced by these theoretical backgrounds, aims to contribute to this important and necessary multidisciplinary research between environmental sciences, art and social sciences.

This section presents an overview on the state of the art of ES and NBS, exploring their intersections with art, science and social engagement. Section “Methods” outlines the project framework in which the Aula Verde has been conceived, and describes the methods used for its design, implementation, and ES assessment. This paragraph also outlines the public participation actions and communication activities undertaken as part of the project. Sections “Results” and “Discussion” will respectively present and discuss the findings of the project, including the delivery of cultural and regulatory ecosystem services by Aula Verde Aniene, the social impact of participatory activities, and artworks arising from the multidisciplinary research. The final section of the discussion endeavours to relativise the ES framework assessed for Aula Verde, going beyond the ES description in numerical terms and introducing the shinrin-yoku as a practice to reach a more complete people’s awareness on nature elements. The conclusion section summarises the project’s contribution, reflecting on its wider implications.

### Nature-based solutions and the related ecosystem services in urban environments

To spread the use of NBSs and to guarantee social justice in terms of equal access to nature in cities, it is crucial to communicate the importance of NBSs to policy-makers and to the public. The concept of ecosystem services (ESs) has been developed for such purposes to assess the benefits that flow from nature to people. Following the Millennium Ecosystem Assessment reports, ESs can be categorized into provisioning, regulating, life-supporting and cultural ESs. Natural ecosystems are multifunctional, and they can provide a wide range of services simultaneously. These benefits are intercorrelated and difficult to quantify and fully evaluate. The most recent European Union classification on nature-based solutions identified 12 societal challenges that could be addressed by NBSs, for each of which key performance indicators have been described. The indicators to be used for ES assessment remain a much-debated issue^21^. Furthermore, difficulties persist in balancing the relationship between ecosystem services and human needs^21,22^.

NBSs in urban ecosystems supply regulating services such as climate control and carbon sequestration, temperature regulation, purification of air, water and soil, flood control, and life supporting services such as oxygen production and nutrient cycles. A third category of ES, very relevant in urban environments and usually less evaluated than others, is represented by cultural ecosystem services (CESs)^23^. CESs are nonmaterial and/or socioecological benefits that people obtain from contact with ecosystems^24^. The general dependence of CESs on an individual’s value systems makes their assessments less quantitative than other services^25,26^, and the complex relationship existing among CESs often leads to overlapping and double counting^27^. The most frequently evaluated CESs are recreation, ecotourism, and aesthetic values, while spiritual, educational and research services are less frequently considered, as well as the contribution of green space to inspiration, social relations, cultural heritage, sense of place and cultural diversity^22,25,27^.

Although a high recognition is granted to the importance of NBSs for the physical and mental health of the population^28^, relatively little attention has been given in the field of ecosystem services to the ways in which natural experience directly affects human physical and mental health^23^. However, a growing body of empirical evidence is revealing the value of natural environments for the psychological and physiological health of citizens, and a new medical science called forest medicine has been established^1,29,30^.

During the COVID-19 pandemic, evidence has been reported on the significant improvement of urban residents’ health status by visiting urban green areas^31^.

As an exhaustive assessment and quantification of benefits that people gain from being in contact with nature cannot be achieved, it should be considered that the measurable benefits account only for a small fraction of the whole dependence of human existence on natural ecosystems, taking into account that humanity depends on a healthy environment^26^. However, part of the society seems to be still unaware of this correlation.

To raise public awareness of environmental issues and of the pivotal role of nature in facing them, it is very important that scientific concepts are made public and readily comprehensible. The lack of public awareness and support has been identified as one of the most influential barriers to NBS uptake and implementation^32,33^. The fact that this aspect is often neglected by the scientific community reduces the impact of scientific research itself.

In the present manuscript, the authors propose a pilot project linking art, science and socioecological themes in a participatory path of knowledge.

### NBS, art and social engagement

The NBS seem to gain strengthen when coupled with participative processes. The theme of collaborative approaches to governing NBS has been theorized starting from 2019 and co-creation processes have been described in scientific literature reaching a pick of publications in 2021 (data based on the results obtained through the query on Scopus “nbs AND co-creation OR participative OR co-governance”). This relatively new concept offers a range of benefits for the implementation and effectiveness of NBS. First, by engaging more diverse actors, urban planning can enhance the pool of available competencies and benefit from wider perspectives in green spaces planning to more fully leverage the potentials of NBS^34,35^. Second, co-creation and genuine participation can be powerful tools to ensure the relevance and acceptance of NBS. As the acceptance of infrastructure developments is determined to a considerable extent by public attitudes, ignoring or mishandling public opinion could lead to significant criticism and may even lead to project abandonment^33,36^. A further benefit of the co governance of NBS is that such approach can contribute to social engagement and inclusion, also dampening the influence of powerful lobbyists interests^37^.

The nexus between art, NBS and social engagement, although neglected in past years, is getting strength thanks also to the New European Bauhaus initiative, proposed as a bridge between the world of science and technology, art and culture^38^.

One of the few case-study in which the art vision supported the NBS co-creation was described by Alméstar et al.^35^. In this project several NBS prototypes were developed by multi-stakeholder working groups led by different artists. The authors critically analysed the transfer knowledge among actors and systems involved in the co-creation sessions through conceptual maps. The artistic approach in this case-study allowed participants, coming from different social systems and diverse forms of knowledge, to collaboratively build unexpected results, making for proposals that would be difficult to obtain from a linear and purely scientific approach^35^. This study, even if it only presents the NBS design phase, opens interesting lines of research wondering what the required conditions for a successful co-creation process in the field of art/science are.

Another project carried-out in the city of Turin (Italy) and described by Dogan et al.^39^ combines art and nature in NBS design to improve the environmental and economic conditions of the place, stimulating art-related tourism. Furthermore, the authors stated that the regenerative art practices in shared spaces play a crucial role in community engagement.

Herrmann-Pillath et al.^40^, as already anticipated by Mel Chin and Andreco artworks^41–43^, surpass the notion that constructing NBS in an artful way entails applying aesthetic principles in its design to raise awareness and promote dissemination. Developing the notion of “biotic artworlds” by Richard Prum’s^44^ and of “more-than human art” inspired by Dewey^45^, the authors perceive art as the key medium of creative agency of humans interacting with nature. In this way, the functional requirement of NBS can be fulfilled by conceptualising NBS design as artwork^40,43^.

The Aula Verde put into practice these principles, it involves art not only as a means to raise awareness of NBS, but it is an artwork in itself. The combination of contemporary art practices with NBS is an innovative approach for both research fields. Artistic language provides a more poetic perspective on NBS, helping to build a new imaginary in the humans and to reconnect them with the non-human. At the same time, NBS gives more scientific strength to environmental art theories. The combination of these practices fosters the participation of the inhabitants that are more emotionally involved and reconnected the intervention space and the wider ecosystem.

### Art, science, and socioecological themes in the climate crisis

Art, science, and socioecological themes have been interconnected in Europe since the rise of natural history study in the eighteenth century. At the dawn of modern science, the study of plants and geography was correlated by drawings with an artistic interpretation, as we can see in the illustrations of the Swedish botanist Carl Linnaeus, the Dutch zoologist Albertus Seba and in the surveys of the German geographer Alexander von Humboldt. Science has elaborated its claims through the representation of geological formations, maps and organisms.

From the eighteenth-nineteenth centuries, a romantic approach spread across the arts in literature, painting and sculpture. The poets of nature, such as Wordsworth, Coleridge, and Blake, showed a more visionary and mystic nature. In painting, the representation of the sublime of nature as well as the “uncanny” (*Unheimlich* in German) of the wilderness presented a challenge to the order of the eighteenth century scientists. By the end of the nineteenth century, this approach to Nature became more political, a direct confrontation with the effects of the Industrial Revolution. Intellectuals and artists started to underline the dichotomy between humanity and nature in influential novels such as “Walden or life in the woods”^46^, “*L’homme et la terre*”^47^ and “Fields, Factories and Workshops (Tomorrow)”^48^. Visual art was also influenced by those cultural debates.

By the 1960s and 1970s, another strong ecological movement arose. Rachel Carson’s book “Silent Spring”^49^, which was the first to demonstrate the dangers of Dichlorodiphenyltrichloroethane (DDT) for ecosystems and social systems alike, marks the beginning of a strong environmental movement. In the 1960s, the emergence of Land Art was a clear sign of the intertwinement of art and nature. The Art in Nature, the Nature in Art was the title of the Biennale of Venice of 1963. In the 1960s, the first site restoration projects, also known as “reclamation art”, took place^50^. The Arte Povera movement^51^ with artists such as Penone, Merz and Acconci in Italy and the social sculpture of Beuys^52^ in Germany also had a strong approach in the use of natural material.

At the beginning of the 2000s, another wave of ecological ideas spread with the rise of the “No global” movement, as people organised international solidarity with the Global South and against the neoliberal global economy. The movement criticised the exploitation of the persons and the natural resources of the south of the world for the interest of the occidental countries. Intensive agroindustry was radically criticised in the name of biodiversity preservation^53,54^. The need for an international environmental right was disseminated by the movement, the movement looked to indigenous cultures where the natural cosmogony orients political and social lives towards a consciousness of the planet, and the movement gathered strength. In the scene of visual art, nature and plants are again protagonists. The contemporary visual culture necessitates a “radical aesthetic reconfiguration to emerge along with new philosophical frameworks”^55^.

In recent decades, especially after the COVID-19 pandemic started in 2020, ecological debates have arisen once again. This time, the debate is ruled by strong scientific influences on art. The IPCC reports, climate movements and independent studies affect visual arts, which took part in the debate on global ecological, sanitary, and social crises.

In the field of art and ecology, Studio Andreco, in collaboration with the association Climate Art Project, cultural and scientific partners developed the Aula Verde concept. This initiative took place in the context of the Climate Art Project (CAP), an itinerant, multidisciplinary project between art, science and environmental activism, conceived by studio Andreco, inspired by the latest scientific and social research on climate change^42^.

As Serenella Iovino affirmed, Natureculture is at the core of Andreco’s works. At centre stage in his eco-artistic and scientific research we find the human impact on natural systems, the sustainability of our footprint given the planet’s limited carrying capacity, the way we shape the environment, and a host of symbols representing our relationship with the more than-human world^56^. The CAP project started in Paris in 2015 during the Cop21 conference on climate change, the Paris agreement and the global climate march. The Climate Art Project is composed of a series of interventions that took place in different cities worldwide. The aim of the project is to raise awareness of global warming and to disseminate NBSs as best practices for climate change adaptation and mitigation. The CAP involves actions for Climate Justice and Social Justice. CAP collaborated with institutions, local, national and international agencies, research centres, universities, cultural operators, museums, art galleries, foundations, environmental associations and groups of citizens, realising local intervention with an international perspective. Through visual artworks based on scientific studies and the active participation of the communities, CAP contributed to the debate on the environmental transition and ecocriticism^56^. At each site where CAP operated, it highlighted the different issues affecting those territories and the possible climate actions to be undertaken. In Portugal, the main themes were heat waves and wildfires, in Puglia, the main theme was the desertification caused by rising temperatures, in Venice, the main theme was sea level rise, and in Delhi, the main theme was air pollution and the condition of the Yamuna River. Visual art, environmental humanities and political ecology are disciplines that are discussing possible ways out from the ongoing climate and social crisis^57^.

**Figure 2:**
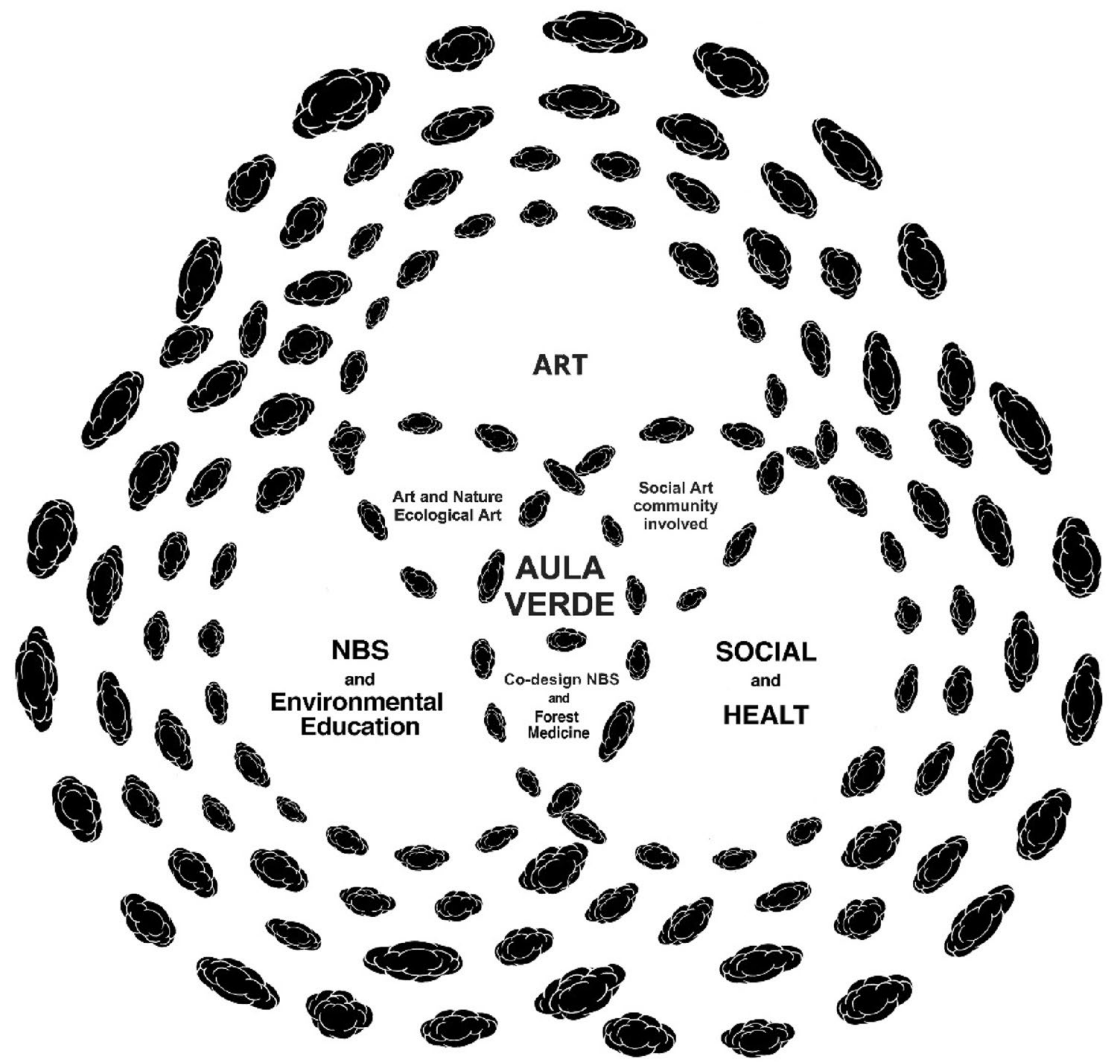
Graphical artwork by Andreco (Andreco Studio) that summarises the concept of Aula Verde, highlighting its interdisciplinary aspect (*Aula Verde* = *NBS* + *Land Art* + *social intervention* + *physic and mental health).*

Aula Verde has been conceived as a local project of CAP and comes from this cultural background. As described in the following section, and in the Fig. 2, Aula Verde has been theorised merging scientific, artistic, political and philosophical theories.

## Methods

The research design was initially developed by an environmental science researcher and an artist. Subsequent to this, an architect and a local citizen’s association were involved in the design and implementation of Aula Verde. To support the data collection and assessment of the ecosystem services related to Aula Verde, a forest ecologist and a research communication expert were later engaged. Most of these actors are authors of the present manuscript. However, an art theorist and an expert on forest medicine contributed to the writing after the project ended, with the aim of providing a more comprehensive, critical, and objective perspective on the work.

Our project involved a diverse group of contributors, each with their unique backgrounds, which illustrates our commitment to interdisciplinary collaboration. This approach is essential for overcoming the challenges associated with NBS implementation. NBS necessitate a multifaceted perspective that encompasses various fields, from environmental science to art, architecture, and more. Our collaboration seeks to break down traditional boundaries between these disciplines, fostering a co-creative process to develop more effective, holistic solutions for both the environment and society.

In this section, the design, realisation and ES assessment of the Aula Verde are described. To contextualise these activities, the principles and definitions of the Aula Verde and its project framework are summarised below.

“Flumen, Climate Actions for parks and rivers” is a multidisciplinary project of art, science and environmental and social activism, focusing on rivers and riparian green spaces.

In the frame of the Flumen project, the first pilot of Aula Verde was realised. Flumen has been conceived as a format replicable in other urban riparian areas.

Aula Verde, or “tree room” in English, is an NBS, a Land Art piece, a place to meet and socialise.

Aula Verde is an innovation in the field of urban forestry because it combines ecology and ecosystem services with public health, social, and artistic visions. Aula Verde can be conceived as a particular way of designing green infrastructures that includes artistic, social and ecological behaviours (Fig. 2).

Aula Verde is a format replicable in different sizes in other sites, always realised by involving local communities with collective performance. The first Aula Verde, named “Aula Verde Aniene”, was realised within the Flumen Project and was designed by Andreco Studio.

The following actions characterised the work approach of the Climate Art (CAP) and Flumen projects.

- Building a heterogeneous network of partners;
- Organising public excursions, walking on the city green areas and along the urban stretches of rivers;
- Designing and realising environmental monitoring of air, water, soil and plants involving students and citizens from all ages. Two modalities have been adopted:
  - Sampling environmental matrices with citizens during workshops from a citizen science perspective;
  - Studying the historical dataset of ARPA, the local environmental protection agency partner of the projects.
- Producing artworks, exhibitions, performances, and installations based on NBS principles. Flags and textile art have been created, together with wall painting, collective performances and plant installations;
- Organising debates, educational-outdoor classes, talks and workshops;
- Using photos, videos and the data-driven images of the project and creating a mediatic campaign to communicate and disseminate the project contents;
- Using contemporary art to create environmental advocacy;
- Connecting with stakeholders and subjects who are interested in developing climate actions and support them with an artistic, social and scientific perspective.

### Design and implementation of the first Aula Verde

Aula Verde is made by concentric circles of trees; plant species are selected according to the specific ecological conditions and criticalities of the site. A circular space is left in the middle of the Aula Verde and becomes the common space accessible for all. The trees composing different circles are staggered to give the perception of being in a room made by trees. The design is inspired by the pattern created by the vegetative propagation of poplars under natural conditions, where the parent tree produces root suckers following a radial scheme^58^. Adult root suckers are arranged in a circular stand to catch the maximum amount of light radiation.

Aula Verde Aniene was created on April 17th, 2021, in the Aniene Nature Reserve in Rome (41° 55′ 54″ N, 12° 32′ 51″ E), following a long participatory process that involved scientific researchers, activists and citizens of all ages through workshops, performances and debates.

The name Aula Verde comes from the shape of the work, made up of poplar and willow trees, *Populus alba* and *Salix alba,* arranged on two large concentric centres with a diameter of forty metres. The work will take shape over the years and growing it will return innumerable benefits to the territory. Some of these ESs have been estimated and forecasted, as described in the next section.

The number of trees composing Aula Verde Aniene is limited, and the design is simple, as it can be readily perceived by citizens. This stand of trees has been conceived as an example of an NBS, where the related ESs can be easily assessed and described to citizens in a clear and understandable way.

**Figure 3:**
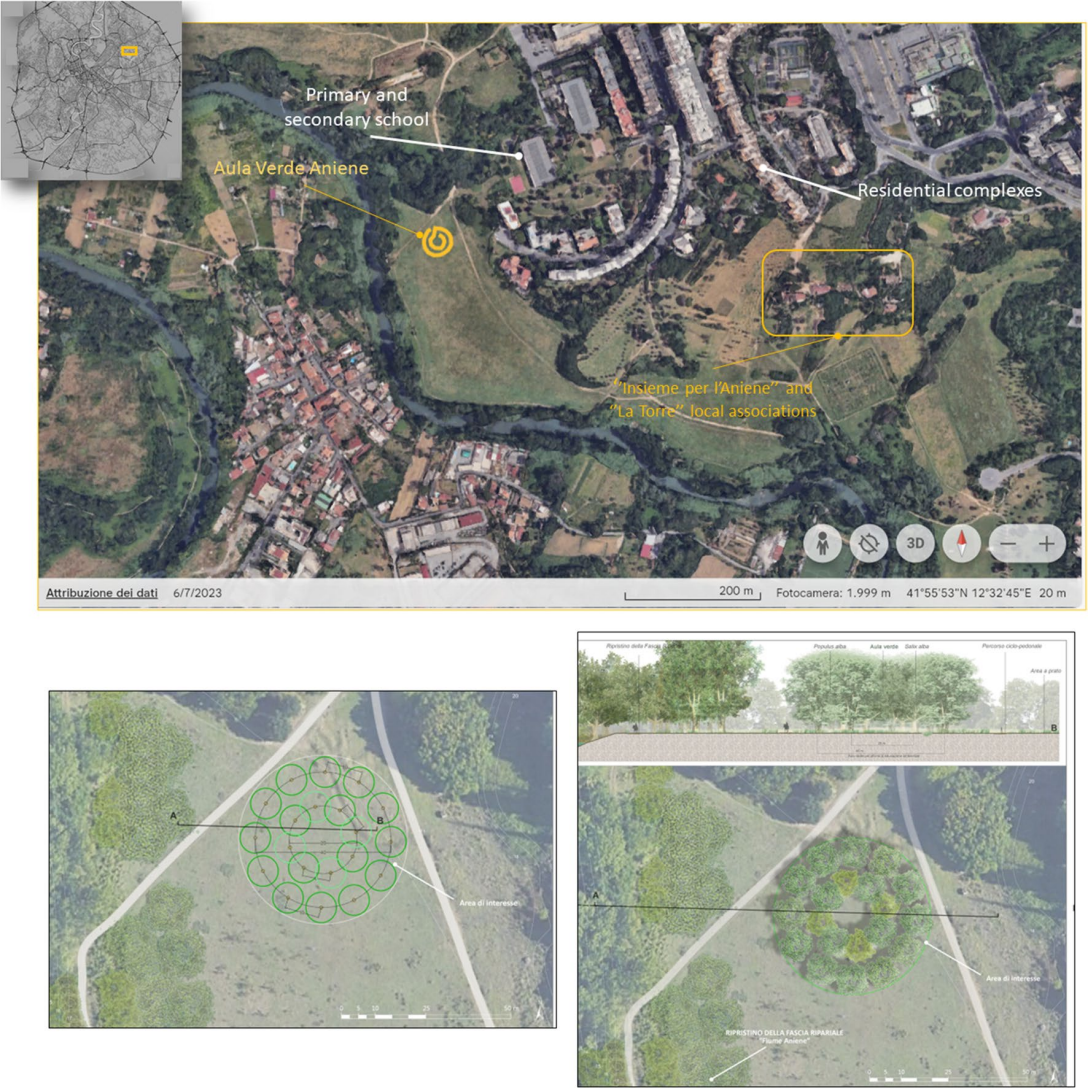
The first picture is a satellite image from Google Earth software showing the location of Aula Verde and nearby citizens’ utilities. The second and third images show the project of Aula Verde Aniene and have been extracted from the rendering of the Aula Verde project design prepared by the architect in charge of the regulatory procedure for Aula Verde implementation.

The location of the tree stand was chosen in one of the lower positions of the park where the rainwater naturally accumulates (Fig. 3). The site, approximately 150 m from the river Aniene, is flooded during periods following intense precipitation events; for this reason, Aula Verde Aniene has a role in intercepting subsurface water flows before reaching the water body. Additionally, the location was chosen because it is near a walking path that connects the park with a nearby school and other public areas of the natural reserve.

The project design and the related documents have been presented to the Regional Authority Administration for risk management evaluation and permission procedures.

The tree planting was performed in a collective manner involving citizens. After planting, participants were asked to fill out a form with information on each tree (size, species, health conditions). The collected information was used as data input for the Aula Verde ES assessment.

Aula Verde Aniene represents a pilot study; based on the same scheme, other similar projects of Aula Verde have been realised in Italy, following a site-specific design, depending on the environmental, climatic, and social contexts. The present manuscript focuses on Aula Verde Aniene realisation. Table 1 summarises the main characteristics of each Aula Verde realised to date to contextualise the first pilot study.

**Table 1:** Projects of Aula Verde realised between 2021 and 2023 following the first scheme of Aula Verde Aniene.

Name	Location	Habitat type	Local peculiarities	Specific ecosystem service addressed	Plant species composition
Aula Verde Aniene	Rome, Nomentano -Monte Sacro district	Cultivated area of gardens and parks (riverside, seasonally wet grassland)	Nature reserve in a dense urban area near schools	Water purification	*Populus alba, Salix alba*
Aula Verde Lago Ex-Snia	Rome, Prenestino-Labicano district	Cultivated area of gardens and parks (lakeside)	Small community self-organised green area in a dense urban area	Local climate regulation	*Populus alba, Populus nigra, Salix alba, Salix japonica*
Aula Verde XFarm	San Vito dei Normanni, Brindisi province	Arable land	Intensive olive cultivation in arid environments. The olive trees of the region have been subject to a widespread infection by Xylella *fastidiosa* bacteria	Pest and disease control and biodiversity	*Ceratonia siliqua, Cydonia oblonga, Sorbus domestica, Crataegus azarolus, Ziziphus sativa, Punica granatum, Arbutus unedo, Mespilus germanica, Ficus carica var. caprificus*

The habitats were identified following the EUNIS Habitat Classification.

**Figure 4:**
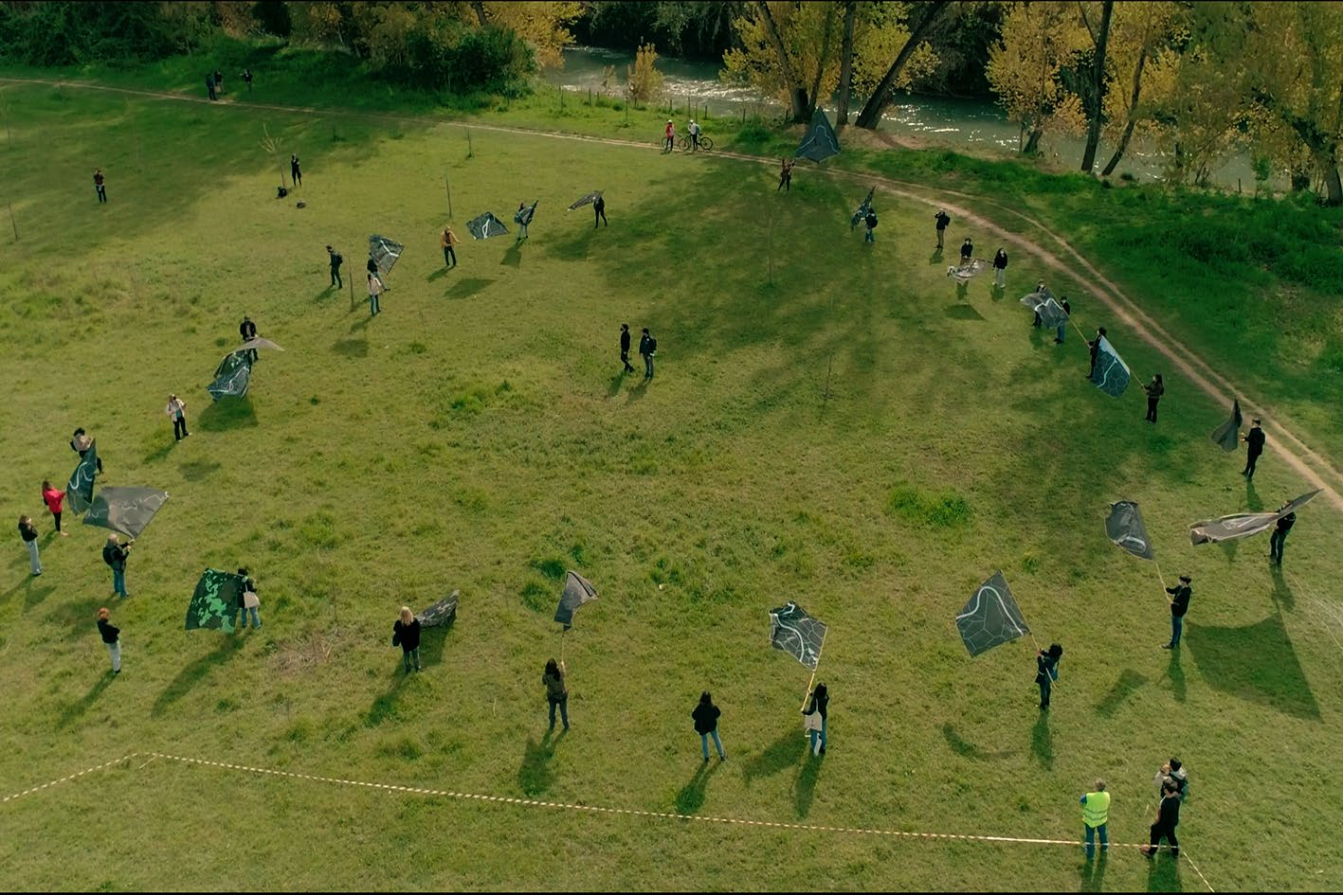
Collective performance organised for Aula Verde Aniene planting. Photo author: Futura Tittaferrante.

The distinctive factors characterising Aula Verde projects are as follows:

- The plantation follows a circular pattern;
- The selected plant species are among the most efficient in mitigating the critical local environmental issues;
- Local communities and citizens are involved in plant maintenance and ES monitoring;
- Collective performances are organised for the realisation and the inauguration of the Aula Verde (Fig. 4);
- Photo and video documentation of the performance to reach a widespread diffusion of the activities and an international dimension;
- The design is based on a site-specific format aimed at highlighting different functions of trees in anthropized environments.

### Ecosystem service assessment

Regulating ecosystem services were assessed through participatory data collection on the trees that comprise Aula Verde. Survey questionnaires and interviews were used to monitor cultural ecosystem services, highlighting people’s preferences and perceptions. Additionally, the quantitative calculation of event participants and social media-based data were utilised to evaluate the impact of the Flumen project on the community. The methods used for the surveys are detailed in the forthcoming section.

The data collected from each ES evaluation method were processed to assess different regulating and cultural ES indicators linked to Aula Verde (row data available in SM 4, SM 5, SM 7, SM 8, SM 9, SM 10). These indicators include carbon storage and sequestration, air pollutant removal and prevention of surface runoff and the rate of perceived significance of CES and health benefits. The data from interviews and questionnaires were collected and processed through Google-form tool and Microsoft Excel™ application. The results were elaborated to evaluate: (i) the level of awareness of the citizens on the ES related to trees; (ii) the main CES provided by the Aula Verde as perceived by the citizens.

i-Tree Eco software, version 6, was used to assess a set of regulating ES related to the presence of the trees composing the Aula Verde Aniene. i-Tree eco is a model that uses tree measurements together with weather and pollution as input data to estimate ecosystem services and structural characteristics of the urban forest. The software suite has been developed by the USDA Forest Services in collaboration with numerous public and private partners. Since 2006, it is in the public domain and available by request through the i-Tree website. Due to the user-friendly approach of this model, it has been widely used in case studies across the world by communities, nonprofit organisations, consultants, volunteers, and students to report on the urban forest at all scales from individual trees to parcels, neighbourhoods, cities, and entire states. By understanding the local, tangible ecosystem services that trees provide, i-Tree users can link urban forest management activities with environmental quality and community liveability^59^.

Part of the input data were collected during the leaf-on season by the citizens attending the participating planting event organised within the project (figure in SM 5). Each plant was identified with a number, and after a short training course, the participants were asked to fill a printed form collecting the following features for each tree specimen: tree species, diameter at breast height (DBH), total height, crown condition, crown top and base height, crown width, crown light exposure, distance to the buildings, and signs of stress. All the field data were subsequently recorded in an Excel file and imported into the i-Tree Eco model.

Local air pollution and meteorological input data were derived from a local dataset recorded during 2015 at the meteorological station of the Urbe Airport (Rome), 4 km from Aula Verde. Plot data are shown in the supplementary material.

Based on the above, ecosystem services related to the urban forest Aula Verde have been assessed, such as carbon storage and sequestration, air pollutant removal and avoided surface runoff.

Considering the young age of the plant, the forecast function of i-Tree eco was used to estimate the evolution of the accounted ecosystem services over the next 50 years. i-Tree uses structural estimates, environmental and location variables, species characteristics and growth and mortality rates to forecast future tree DBH and crown size. Forecasted benefits are then estimated by the model based on the projected tree growth and leaf area.

Further information on the model equations is available in Nowak 2020^60^.

The results of ES assessment were presented in the present manuscript as numeric indicators and graphs, row data are.

### Public participation actions and communication activities of the Aula Verde project

The Aula Verde has been conceived as an artistic intervention, an NBS and an aggregation place in which people can experience the wellness derived from trees, nature, green, natural sounds and temporary suspension from the city. The involvement of the people is a necessary process to give the right value and meaning to the room. Public participation has played a pivotal role in the creation of the Aula Verde and in all the actions organised in the frame of the CAP and Flumen projects. Different kinds of actions have been performed, some of them not in the Aula Verde or not strictly dedicated to it, but all the events have signed a path towards the consciousness of their implication for environmental enhancement and social wellbeing. The participatory process involved scientific researchers, activists, and citizens of all ages. It is possible to summarise the public participation in four types of actions strictly interconnected as follows.

#### Workshops

A series of workshops were organised in different locations, starting from March 2020 to June 2022. Twelve of them took place at the Nature Reserve of Aniene Valley (Rome), with the collaboration of the association “Insieme per l’Aniene”, local partner of the Flumen project. Most of the workshops were promoted through the press, social networks and mailing lists. Three events were specifically addressed to students from lower secondary and secondary schools of Rome. Each workshop focused on one environmental issue or one aspect of NBSs. Citizens were involved in water sample collection from an urban river (Aniene) and analysis with the support of experts from ARPA Lazio (Regional Agency for Environment Protection), in a rapid assay with *Lemna minor* (duckweed species) as a water quality bioindicator, in the assessment of the eco-physiological state of trees forming Aula Verde, in collecting data of each tree specimen and in discussions about the ESs that are derived from the tree stand.

Two days were spent with citizens along the banks of the Tiber River in the centre of Rome to debate the role of the green areas in the city, to map the spontaneous riparian macrophyte, and to compare plant cover and species distribution with previous studies in the same location to highlight changes over the last 100 years. The event has been organised in collaboration with Roma Tre University, a scientific partner of the project, and together with local associations involved in river monitoring.

#### Participatory planting

On the 17th of April 2021, the Aula Verde was realised through a collective performance involving citizens (Fig. 4 and SM 5). Flags representing the area of the intervention, the river Aniene and riparian green spaces, were given to the participants. With a slow, ritualistic parade, the citizens moved to the location of the planting, where after a simple choreography driven by the artist Andreco, people were introduced to the sense of the operation and the principle behind the Aula Verde and planted the trees composing it. Each participant completed a form with data and information on the tree he or she had just planted. On Earth Day 2021, 22nd April, a video telling the participative planting was published and shared through the principal social networks (Video 1, https://www.climateartproject.com/2021/04/20/aula-verde-climate-action-and-art-project-by-andreco-for-earth-day/).

#### Public debates

Three public debates were promoted from September 2020 to March 2022 in three different locations. The first one took place in the “Fondazione Baruchello”, an art foundation of the artist Gianfranco Baruchello, with the support of the art historian Carla Subrizi. The activity of Baruchello has been focused on the relations among art, culture, philosophy, and nature since the seventies^61^. The event was presented via streaming with only the organizers present, due to the pandemic. After lectures from artists, gallery curators, researchers and professors, the citizens were invited to discuss and contribute to the actions needed for the success of the project Aula Verde. The Aula Verde project has been compared with the Agricola Cornelia realised by Baruchello^62^. In May 2021, a talk was held in the Roman museum “Palazzo delle Esposizioni” with all the scientific partners and associations involved in the project for a multidisciplinary comparison and exchange of competences regarding scientific results, planting of the Aula Verde and phytotechnologies related to it. The event was also made available to the public by streaming on social networks.

On World Water Day 2022 (World Water Day was established by the United Nations in 1992), Museo MAXXI in Rome hosted a debate articulated around the Flumen project, the results, and the effects of the Aula Verde on the environment. The video Tiberina, a documentation of the performance directed by Andreco and dedicated to the river Tiber, was also projected. This debate took place in presence.

#### Surveys

Three different surveys were carried out during the project events to evaluate the awareness of the public about the status of the rivers and the presence of green infrastructures in Rome. An external group from Sapienza University has been engaged in it and was also charged with the ex-post assessment of the events organised within the project. For two of the three surveys, the number of participants was not sufficient for an extensive evaluation of the responses, but the results were still considered for internal feedback. The third one was proposed to a wider and more differentiated audience, through contacts of the Insieme per l'Aniene association and the La Torre social centre, two realities that are located in the park and experience it on a daily basis.

A specific question was designed to assess the level of awareness of citizens on ES related to trees, and to identify the categories of ES most considered. This was an open-ended and optional question.

A multiple-choice question was dedicated to the perception of CES and health benefits provided by Aula Verde. Different categories of CES were suggested to the participants based on the MEA classification system^63^ and for each item a rating scale ( 0 for not at all, 1 for average, 2 for very much) was provided to assess the rate of perceived importance attached to each category.

Questions proposed can be found in supplementary materials (SM 3).

## Results

### Regulating ecosystem services of the Aula Verde Aniene

**Figure 5:**
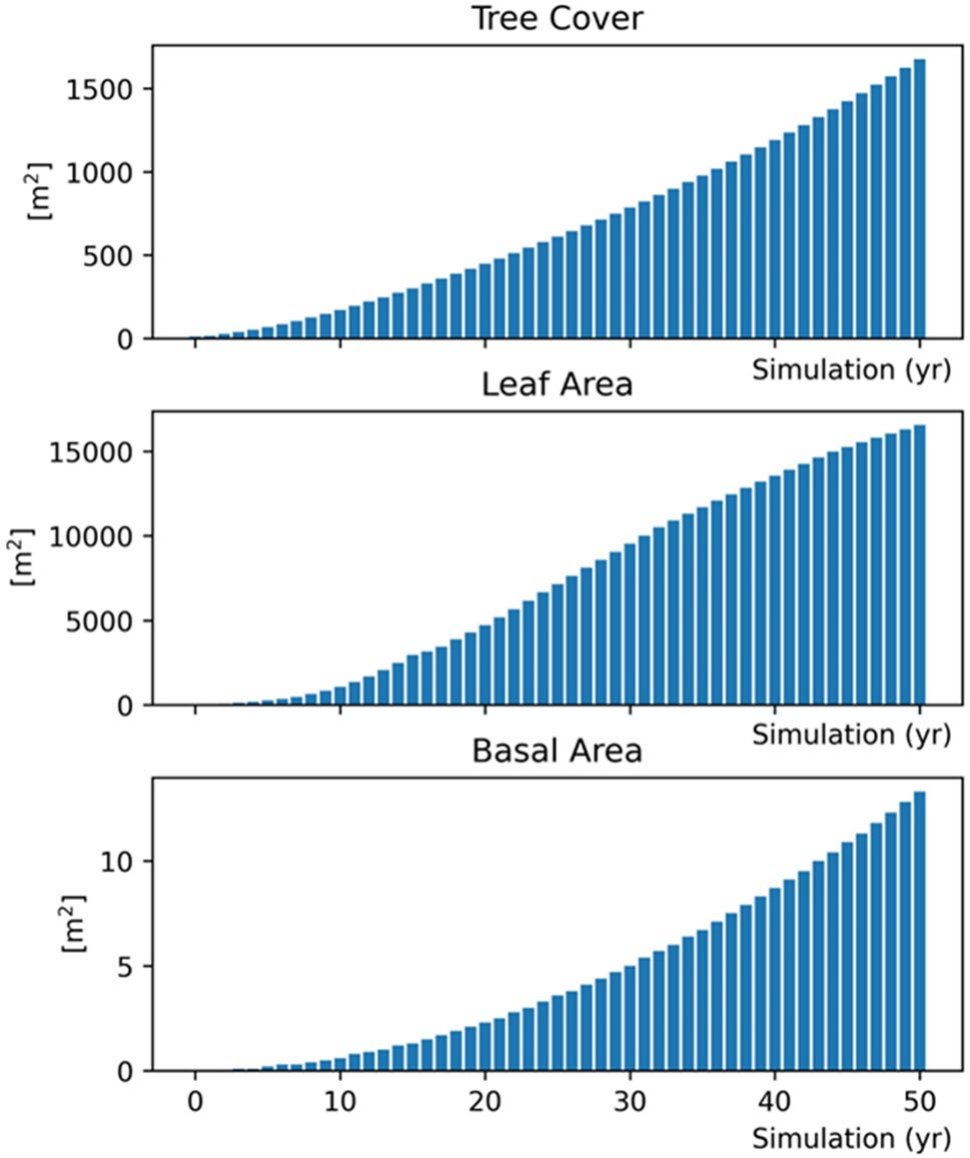
Forecasts of Aula Verde Aniene tree cover, leaf area and basal area estimated up to 50 years after planting.

**Figure 6:**
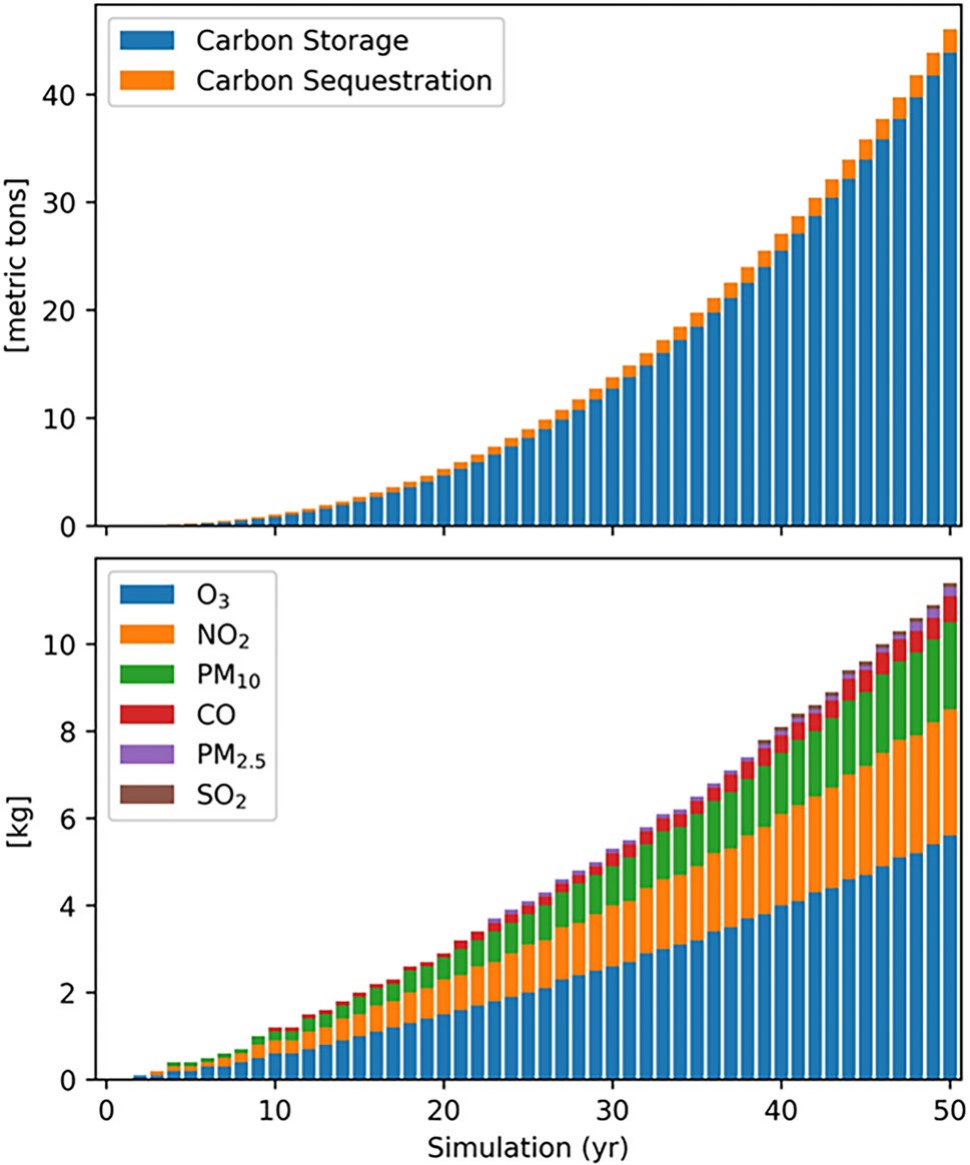
Carbon storage, carbon sequestration and air pollutant removal calculated for Aula Verde Aniene at each year after planting, up to 50 years.

Based on the properties of each tree species and on the morphological characteristics recorded for each specimen, i-Tree equations estimated the whole tree dry weight biomass and carbon storage. Using the forecast function of the i-Tree model, the growth of each tree was estimated based on the health condition and light exposure characteristics recorded as input data and reduced by the amount of carbon lost due to tree mortality and decay estimates. The model estimated tree cover, leaf area and basal area each year, from the first year up to fifty years after planting (Fig. 5). The gross amount of carbon sequestered annually was then calculated by the model from the difference in estimates of carbon storage between the current and the next year assuming standardised growth based on canopy light exposure^64^. The gross sequestration of Aula Verde trees is approximately 11.02 kg of carbon for the first year (2020–2021), and the Aula Verde carbon storage accumulated over 50 years (2020–2070) has been estimated to reach 48 tons (Fig. 6).

**Figure 7:**
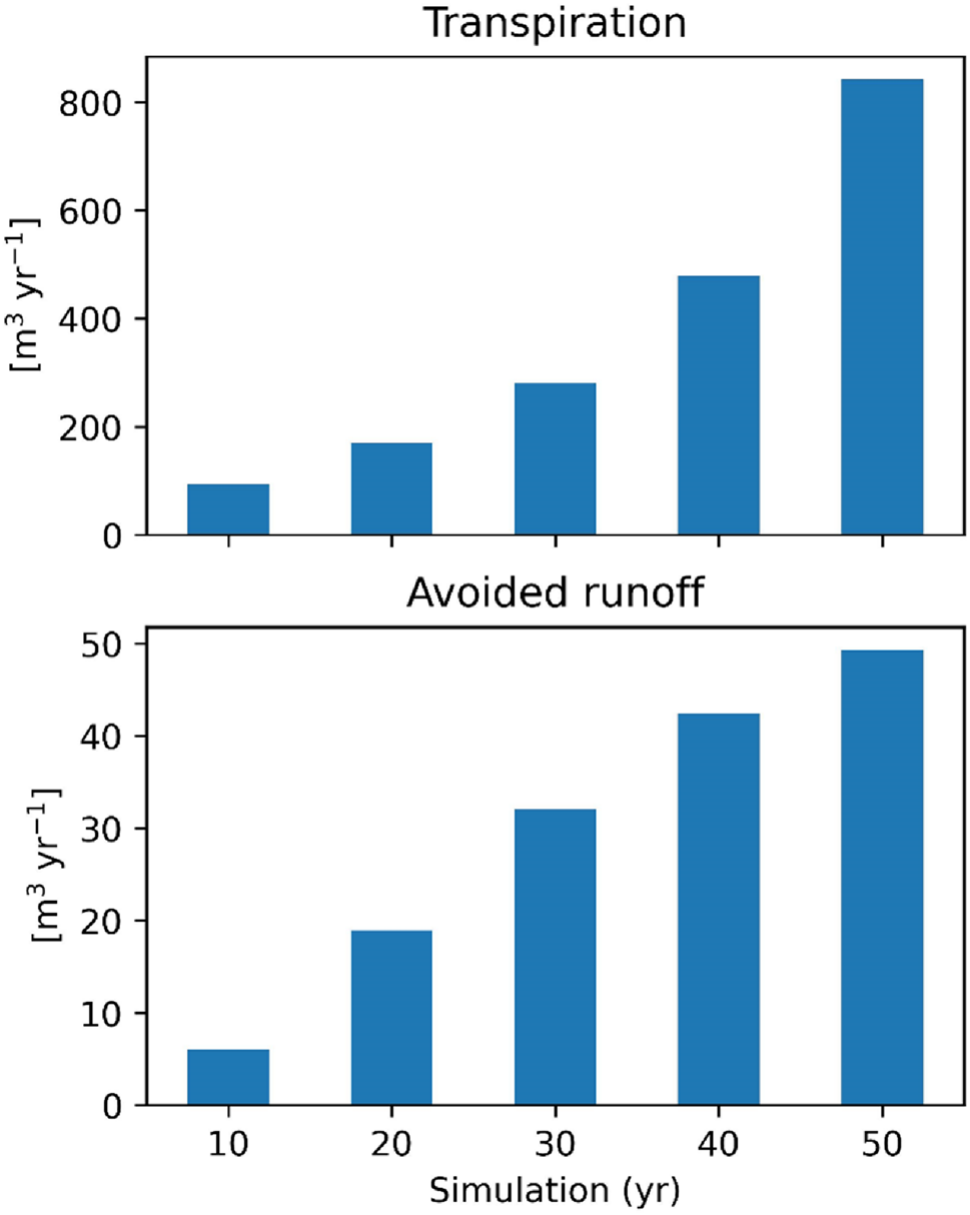
Transpiration rates and avoided runoff estimated for Aula Verde Aniene at 10, 20, 30, 40 and 50 years after planting. The simulation was based on the DBH forecasted by i-Tree eco.

The surface runoff, defined as the portion of the precipitation that reaches the ground and does not infiltrate into the soil, is reduced by the canopies of Aula Verde, which intercept precipitation, and by the root system promoting infiltration and storage in the soil. The i-Tree model calculated avoided runoff based on rainfall interception as the difference between annual runoff with and without vegetation, considering the precipitation intercepted by leaf/bark area and the amount of water that evaporates and transpires from trees. Avoided runoff was estimated at approximately 30 L for the first year, 5 m^3^ yr^–1^ after 10 years from planting, reaching 48 m^3^ yr^–1^ after 50 years from planting (Fig. 7). As i-Tree eco V6 does not include the forecast option for hydrologic outputs, the estimation for each tree age was assessed by performing a single simulation for DBH calculated at 10, 20, 30, 40 and 50 years.

Pollution removal has been calculated for ozone, sulphur dioxide, nitrogen dioxide, carbon monoxide and particulate matter less than 2.5 µm, the most impactful fraction in relation to human health. It has been estimated that Aula Verde trees remove 53.21 g of these pollutants the first year, reaching 11,000 g after 50 years. The greatest portion of estimated removal is constituted by ozone, followed by nitrogen dioxide and PM10.

### Public participation in the Aula Verde activities and cultural ecosystem services

In all the activities performed during the Flumen project, citizens participated with enthusiasm. People attending workshops and debates were informed about scientific and artistic aspects, specifically about NBSs and about the strict interconnections between the environment and human health. Art had a main role in the process of building common awareness of green topics. People were also actively involved in monitoring and planting. In particular, the participating planting in April 2021 represented the most engaging moment in which the people, called to action, had the opportunity to touch with their hands the plants, the soil, the tools of the effort needed to preserve and improve the environmental quality. At the same time, they could experience the pleasure and satisfaction derived from realising a work of art in relationship with nature. Based on the information collected during the participative planting by the project partners and by the external assessment group from Sapienza University, we found that the participants, gender balanced, were unaware of most of the benefits associated with the presence of trees; this was especially true among the younger generations. Most of the participants declared that they had never planted a tree in their life.

Maintenance has been the most critical aspect of the Aula Verde Aniene project, during which 8 trees out of 20 were replaced following drought and grazing events. For the Aula Verde Ex Snia (Table 1), all the trees were in good condition 1 year after planting because there was more constant maintenance made by the citizens, who visit and take care of the park. In both cases, the irrigation system could not be installed, but the results showed the crucial role of the community in taking care of the project after the realisation.

During public events, people exchanged opinions, doubts, and emotions with the experts and each other, demonstrating the intimate urgent need to participate in the process and deepen knowledge, creating a coral voice in support of wellbeing in the natural environment. Contemporary art is a language able to touch people’s sensibility, and an art piece such as Aula Verde represents a new way to reflect on human roles in the environment and to feel nature^43^. Citizens were involved thanks to local associations and to the echo of the project in the media. The Aula Verde, CAP and Flumen projects have been presented through art magazines, newspapers, and Italian national television.

Workshops and other in-person events had an attendance of approximately 30–40 persons each, for a total of almost 700 people involved. Another 150 people attended in presence the debate at the MAXXI Museum. The audience of the Flumen events was predominantly young, highly educated people, which fostered discussion on climate change and ecological issues. For the streamed debates (Fondazione Baruchello and Palazzo delle Esposizioni), the participation was approximately 400 people each. The engagement on social networks (Facebook and Instagram) was very active; the total number of interactions on social networks was almost 300,000. The video of the participatory planting (which was released a few days later, on Earth Day 2021) had almost 50,000 visualisations (Video 1).

Table 2 summarizes the indicators used to evaluate the impact of Flumen project on the community.

**Table 2:** The table summarises the indicators used to evaluate the impact of the Flumen project on the community.

Number of persons involved in the public events on Aula Verde	700
Number of persons involved during “in presence” debate	100
Number of persons involved in streamed events	800
Number of on-line visualisations of Aula Verde video	50,000
Number of interactions on social networks	300,000

The survey proposed through Insieme per l’Aniene e La Torre contacts received 121 replies. The responses are presented as raw data in SM 6. The age group most represented is in the over 56 age group (63%), followed by the 36–55 age group (23%). Notwithstanding a 12% of persons between 26 and 35 years, it is clear that the public of the survey was mainly adult and senior. This could be an effect of the greater availability of the middle-high age group to complete the questionnaire, or it could reflect the actual age composition of park users. Almost 50% of the participants have completed high school and 45% have a degree, so the public belongs to a medium/high level of education. To test whether the participants were aware of the benefits from trees, an open and optional question was proposed: "What are the benefits associated with the presence of trees?", which received 87 responses. The responses are collected in SM 4. The varied responses have been classified in four categories of ecosystem services: supporting, provisioning, regulating and cultural. The category most represented was cultural (40%), followed by regulating (36%), supporting (21%) and provisionning (3%). Mental health relief and aesthetic values were the most cited benefits among CES. For regulating ES, 'clean air' and 'cool shade' were the most recognised benefits. In 7 answers, people used the word "life" to express the benefits received by the trees, without mentioning the processes leading to them.

**Figure 8:**
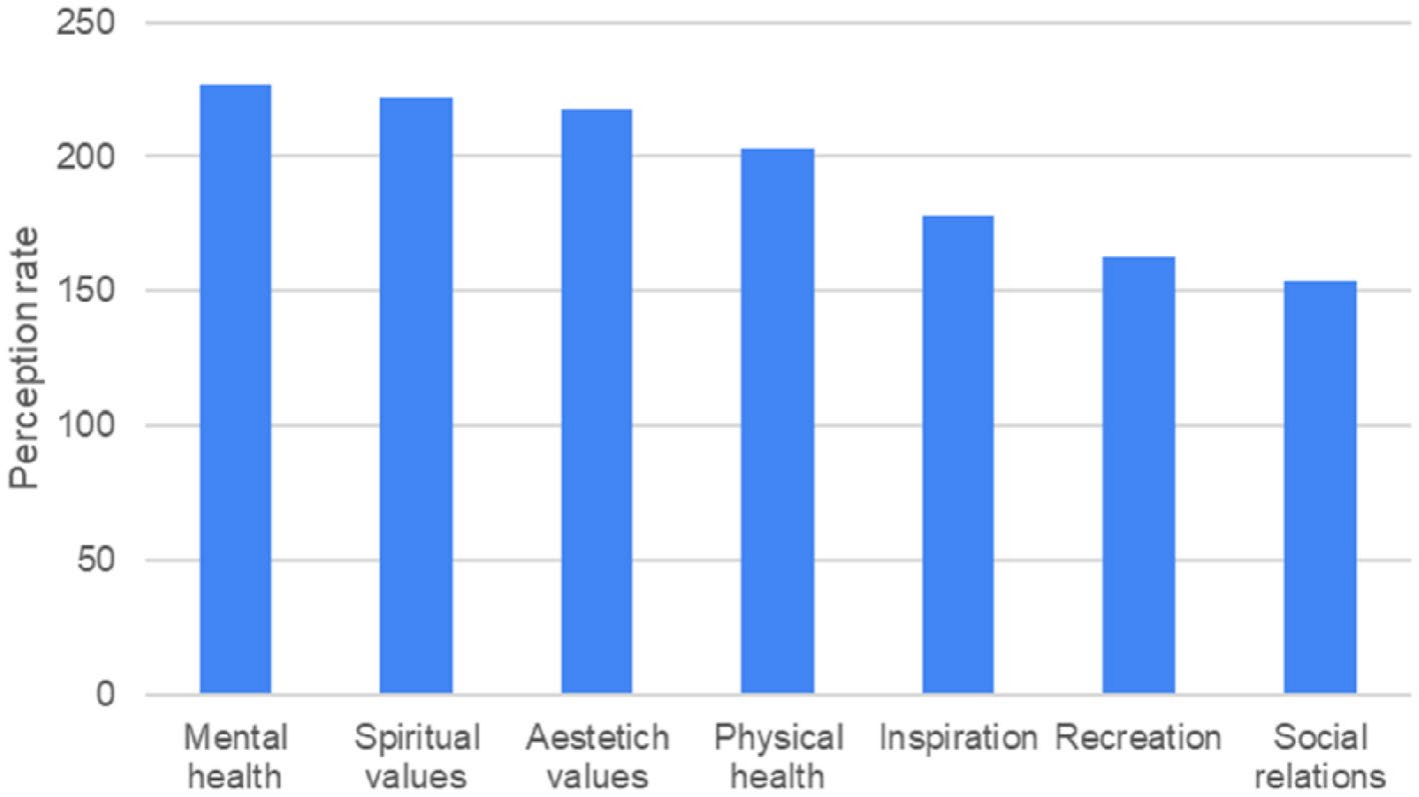
Results of the questionnaire proposed to the citizens’ associations of the park (number of responses = 121). The graph represents the responses collected for the multiple-choice question dedicated to the perception of CES and health benefits provided by Aula Verde. The graph shows the rate of importance (perception rate) given by respondents to each item. The perception rate was calculated as the sum of the rates assigned by respondents to each specific item (0 for ‘not at all’, 1 for ‘average’, 2 for ‘very much’).

Almost the totality of the people involved in the survey and in the events declared to have a sense of well-being in the Aula Verde. The multiple-choice question on the perception of benefits provided by the Aula Verde highlighted that mental and spiritual well-being were the most perceived (Fig. 8). Respondents also reported an aesthetic value of the place and a sense of physical well-being, followed by a sense of inspiration, recreational value and improvement of social relationships.

Of the participants, 93% said that the Aula Verde could help raise awareness of the value of trees and respect for the environment.

### Artwork production

Artworks have been produced as a result of the multidisciplinary research project.

**Figure 9:**
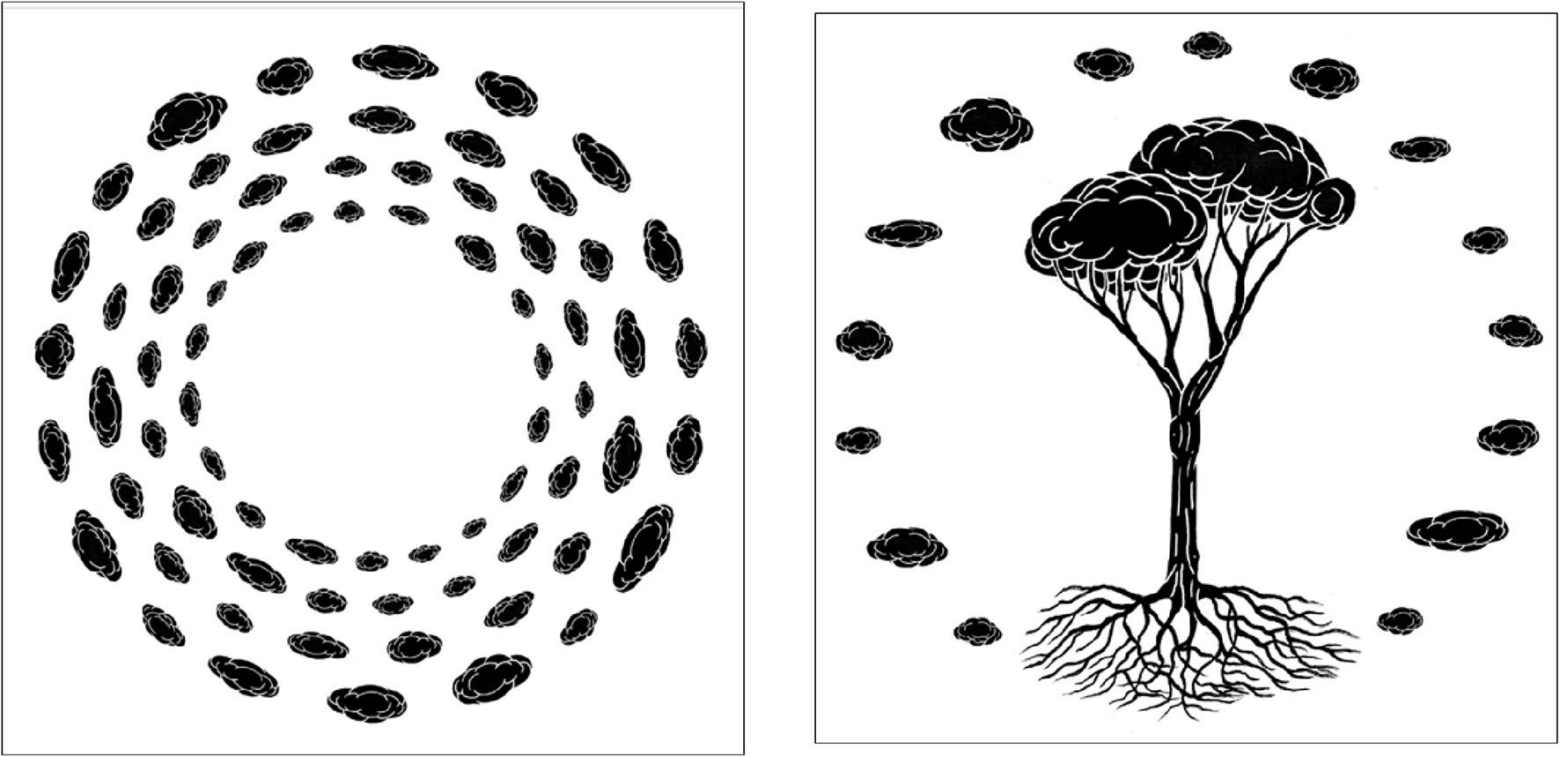
Art drawing produced as a result of the multidisciplinary research project: “Aula verde, Cloud design” on the right and “Aula verde, pino” on the left. Author: Andrea Conte, Andreco Studio.

The Aula Verde Aniene, the Aula Verde Ex Snia and the two Aula Verde XFARM (Table 1) are there as a permanent installation of art in the green spaces, artworks in continuous transformation as the growing trees. Drawings and screen prints have also been realised as an artistic visual interpretation of the Aula Verde (Figs. 2 and 9).

Performances involving the local communities and the project partners have been realised in the tree locations. Flags representing the area of the intervention have been realised and used in the performance. Photos and videos are also art formats that have documented the realisation of the Aula Verde in several locations (Fig. 4 and Video 1). During the period of the dissemination of the project, during the debates at Fondazione Baruchello, at the National Museum of XX century MAXXI, and at Palazzo delle Esposizioni, artworks were presented and discussed.

## Discussion

The Aula Verde Aniene is an innovative and successful experience in terms of public participation and mediatic response, and it promoted a new concept of reforestation that also considers social and artistic aspects. The citizens involved in its realisation were enthusiastic about the experience. Most of them had not planted a tree before. Those people will be visiting the Aula Verde and they will observe it growing during the years. Over time, the Aula Verde will take shape, and the central space will become increasingly isolated from the park outside, giving room to environmental education initiatives, public events, and meetings.

Aula Verde exemplifies the blending of science and art through collaborative co-creation. It underscores the pivotal role of urban green spaces in addressing climate, environmental, and societal challenges, while also providing cultural enrichment. NBS inherently require a multifaceted perspective that encompasses environmental science, art, architecture, citizen engagement, forest ecology, and communication research. The involvement of this varied group of experts and stakeholders not only enriches the project with a wide range of knowledge and skills but also mirrors the complexity of the challenges we seek to tackle in the context of NBS. By fostering this kind of interdisciplinary collaboration, we tried to bridge the gap between traditionally separate fields, encouraging the exchange of ideas and approaches. This collaborative effort is crucial to overcome the obstacles that can hinder the successful implementation of NBS projects and to promote a co-creative process that integrates multiple viewpoints and expertise, ultimately contributing to more effective and holistic solutions for the benefit of the environment and society.

A similar project was carried out in Texas leading to interesting outcomes. The Authors adopted the same multisectoral, collaborative, environmental data-driven approach, involving larger-scale planting compared to the present work^65^. The Texas project built bridges between community groups and other sectors and increased awareness of shared goals, providing a meaningful blueprint for addressing climate change. The results were presented as evidence of the importance of engaging diverse leadership for long-term benefits^65^. In addition to this multi-sectoral data-driven approach, the Flumen project integrated the conventional engineering and environmental perspectives for NBS design with an aesthetic vision.

Aula Verde is also an artwork in the public space and represents a new artist practice in the field of art and ecology. It is an evolution of what other artists did in the past, such as Joseph Beuys, Mel Chin and the Land art artists, connected to the research on NBSs and to the actions to contrast the climate crisis. The use of the circle shape is symbolic, it is present in many cultures and rituals around the globe^66^ and utilised in the agroforestry practices. For example, Yanomami used to have gardens for crop production and social areas in circular shapes in the Amazon Forest^67^. Those circular spaces are itinerant in the forest. The IPCC report on Land Use proposes that indigenous agroforestry is a best practice for land use because it allows food production contributing to carbon sequestration^68,69^.

The introduction of ecological art within the co-creation process can have a significant impact on urban planning, governance, and environmental awareness, by inspiring innovative urban development and influencing policy through the promotion of nature-based solutions^41^. The integration of art into urban green spaces within the framework of NBS presents a transformative approach. This practice involves collaborative co-design, where public art is incorporated to align with the ecological context of NBS. This approach not only enhances environmental and economic conditions but also stimulates art-related tourism, fostering a dynamic urban landscape^39^. The ability of ecological art to raise environmental awareness among urban populations is notable, engaging the public through creative processes and fostering a deeper understanding of ecological challenges, can inspire co-creation strategies to promote "creative energy" in the design of NBS^35^.

Maintenance has been the most critical aspect of the Aula Verde Aniene project, and one-third of the trees have been replaced following drought events. Plant irrigation relied entirely on local associations that were too far from the location to effectively ensure constant maintenance and monitoring (Fig. 3). In Aula Verde Ex Snia (Table 1), the good condition of the plantation after the first year has been due to a more proximal presence of citizens which oversaw its maintenance. These results highlight the importance of a management plan considering social aspects and the crucial role of the community in taking care of the project after its realisation. As stated by several authors, participation in the maintenance phase is fundamental for urban green infrastructures, even if it remains an under-researched field^70–72^.

### Art critics on Aula Verde

The praxes at work in Aula Verde come from a history of ecological art, described in the introduction chapter 1.3. In particular Aula Verde can be considered an evolution of the first practices were site-restoration projects, otherwise known as “reclamation art”^50^. These endeavoured to revive natural sites, particularly those that had been exhausted by strip mining, by turning them into public green spaces. Over the course of the nineteen-seventies, artists were concerned with the aesthetic revitalisation of space through “ecological resuscitation”. To this end, they joined forces with scientists, landscape planners, engineers, environmental specialists, activists, and local communities to create art projects that would overhaul degraded sites. Such works deployed a variety of mediatic techniques not only to restore but also to relay an ecological awareness of the site, including photographs, maquettes, maps, material sediment, scientific samples, data and more.

Aula Verde, compared with the first reclamation art is more influenced from the contemporary research on NBS and climate and social justice. Aula Verde also engages a process of “social sculpture”, a term coined by Joseph Beuys, the intermedia artist and founding member of the Green Party in Germany^52^. Social sculpture endeavours to both teach the public about art and to engage them in the artist’s power of social transformation. In Aula Verde, the artist Andreco calls on a mythic history and brings this to bear on the urgency of ecological care in the present, a time when the world’s waterways are susceptible to biochemical pollution, drought, and flooding. These are the paradoxical times of climate change, which are difficult to understand without drawing from the science that gives a global perspective and the collective will of the public to take responsibility for them. Such actions are powerful, and they look like fiction, science, and protest all at once.

What emerges in the midst of these disparate artistic modalities is a public defence of the living ecology of rivers. As citizens and visitors of the city witness the gathering of people in the public spaces of Rome, moving in formation and flying flags, they are invited to consider the forces that shape and intervene in the environment. These gatherings are political in nature, and they change public spaces through the occupation of a collective organisation. In this respect, the practices at stake in Aula Verde could be considered a form of eco-activism. The artist Andreco also seeks to build public knowledge about the rivers and other urban ecologies; knowledge that crosses the scientific and the social, and which refuses to allow these to be divided into specialised expertise. The actions that would amount to carrying out such responsibility, however, originate from a history of artistic practice. The Flumen project through art aims to create environmental and social agency. For the Flumen project the artist with collaborators and partners organised public walks, collective environmental data sampling and collective performances to transfer a poetic artistic vision and scientific knowledge about the ecosystems to the public. The motivational part is very important for the artist that gives a speech for the participants before the workshops and performances. In the occasion of the realisation of Aula Verde Aniene, part of the Flumen program, the scientific, political and artistic motivation of the project was also shared with the participants. In the months that followed, the concept and motivation of the project were and will continue to be shared by the local partners for each public walk that ended up in the Aula Verde Aniene. A text with the Aula Verde definition and a short explanation of the project is installed permanently in the centre of the Aula Verde Aniene (SM 5).

In the era of climate change, this recourse to the transformative power of art also marks an epochal shift towards an age of political ecology. Political ecology is a concept that informs the ways in which specialised disciplinary knowledge is transacted and geared towards those political concerns that cannot be addressed from any singular epistemological framework but rather needs the diversity of a collective effort. It is in this vein that we discuss Aula Verde across the sciences, the critical history of art, and the politics of climate change.

### Ecosystem services of the Aula Verde and public awareness

The ES framework has been mainly developed as a tool for guiding management and assessment decisions aimed at enhancing human well-being without undermining the ecosystem's long-term productivity^63^. In addition to this function addressed to policy-makers, citizens can also be a target of ES assessment, as people are an important driver of the pattern processes and dynamics of the urban landscape, and the role of civil society is increasingly emerging in support of sustainable planning and management practices^72,73^. Opening people to the full range of benefits through education will increase participation and protection of green space^74^. However, it is crucial to correctly communicate data about ES assessment to citizens, making people conscious of the limits of such an assessment framework^26,75^.

The Flumen project led to the assessment of easily comprehensible ESs linked to the Aula Verde and contributed to spreading curiosity and knowledge about NBSs and related ESs. The project is an example of how scientific concepts can be transferred to communities through art to raise the environmental awareness of the public. Art and science interacted to transmit information to citizens, avoiding oversimplifications and describing life and its complexities^76^. The Flumen project attempted to not restrict the description of the Aula Verde benefits to the four estimated ES indicators, the “take-home message” of the project was rather to show the variety and the wideness of benefits linked to NBSs, considering the reported ES only an example of the complex relations between humans and trees. In fact, citizens must be considered an integral component of urban green infrastructure and, as part of the urban ecosystem, interconnected with all its elements^73,77^.

The people who took part in the survey, which was distributed through the contacts of the local associations, recognised the importance of the presence of trees for citizens. Mental and spiritual well-being, and a sense of beauty are the main benefits identified by respondents, confirming the important role of CES in improving the quality of life in urban landscapes^27,78^. However, the respondents seem to be poorly aware about the specific interactions of trees within the ecosystem. The category of regulating ES was mostly limited to "clean air" and "cool shade", and a number of respondents generally mentioned "benefits for life" to address the multiple ES provided by trees.

The present research adopted the i-Tree Eco tool to translate empirical data on tree characteristics into easily understood, municipal-scale metrics such as tons of pollution absorbed, and C sequestered. The results obtained from the ES indicators used for Aula Verde must be considered as an illustrative part of the assessment; other important elements, such as the soil C dynamics and the energy spent to transport, irrigate, and prune urban trees, have not been accounted for. Considering a larger array of factors, the impact of Aula Verde on human well-being would probably be less evident, at least for the first years after planting. As reported by Pataki et al.^79^, there is an increasing empirical understanding of the limits of tree planting as a solution to climate change and pollution. Urban trees appear to be more promising for climate and pollution adaptation strategies than mitigation strategies^79–81^. Indeed, the adaptation concept has been one of the main messages that, based on the i-Tree Eco output data, the present project attempted to transmit to the public through public events, social media, and press releases.

The Aula Verde represents a paradigm of NBSs to increase the social environmental awareness of the value of trees, to bring citizens closer to the trees, to their processes and to the temporal scale of plants, so different from human time. The forecasted benefits, such as pollution removal, carbon storage and carbon sequestration, estimated for Aula Verde over fifty years highlight how temporal scales heavily influence the extent of the environmental impact of urban trees, as demonstrated by several empirical studies^82,83^.

The i-Tree tool has been used across cities worldwide to evaluate the ES of urban trees and NBSs. While great interest has been devoted to survey methods, software calculations and output data, less attention has been given to the communication and dissemination of the results^84^. In recent years, an increasing number of studies have included social impact and community approaches in i-Tree assessment^85–88^.

Raum et al.^89^ evaluated the use of the i-Tree Eco tool in Great Britain and reported problems with knowledge transfer and exchange among the main barriers that limited the impact of i-tree results on society.

The CES proved to be a relevant component of the ES provided by the Aula Verde; the main benefits of the Aula Verde, as perceived by the survey participants, were related to mental health and spiritual values. Aesthetic values were also the among the most perceived benefits. This last finding shows that aesthetics and NBS are closely linked and that NBS design must respond to needs beyond functionality. The power of a multidisciplinary approach to NBS is that meeting the functional requirements of NBS leads to conceptualising their design as a work of art^40,43^.

### Beyond the ecosystem services of Aula Verde

As already underlined in the present manuscript and widely communicated to the project target groups (such as local communities, students, and citizens in general), the ESs assessed through the i-Tree model and social investigations, represent just the tip of the iceberg in the complex interactions between plants and humans. Such complexity is hardly describable only in numerical terms, and to communicate to citizens all the processes and dynamics involved in this interaction would be even more difficult. Experiencing direct contact with trees, such inside the Aula Verde, can probably communicate this concept more than any other means. Humans have enjoyed green spaces for ages because of the quiet atmosphere, beautiful scenery, mild climate, pleasant aromas, and fresh, clean air. Researchers in Japan have proposed a new and traditional concept called “shinrin-yoku”. Shinrin in Japanese means ‘forest’, and yoku means ‘bath’. Therefore, shinrin-yoku means bathing in the forest atmosphere or taking in the forest through our senses of sight, hearing, taste, smell, and touch, connecting with it^1,2^. Empirically, forest environments may reduce stress and have a relaxing effect; therefore, walking in forest parks has specific beneficial effects on human health. Medical research has tried to correlate forest frequentation with preventive and curative effects against lifestyle-related diseases. Stress and urbanisation are two keywords used to understand the background and needs of forest bathing/*shinrin-yoku*. Based on the above background, a national health programme for forest-bathing began to be introduced in 1982 by the Forest Agency of Japan for the stress management of workers^1,2^. Forest medicine is a new interdisciplinary science belonging to the categories of alternative medicine, environmental medicine, and preventive medicine that analyses the human wellbeing-forest contact nexus^96^. Forest bathing/*shinrin-yoku* has been demonstrated to boost immune function (showing preventive effects on cancers), to reduce blood pressure and heart rate (showing preventive effects on hypertension and heart diseases), and to reduce stress hormones and mental stress (showing preventive effects on depression)^23,91–97^.

Taken together, these findings suggest that, beyond ES, urban forests provide potential preventive effects on lifestyle-related diseases. It is crucial to recover the contact between citizens and trees, educating urban communities about human-plant interactions and introducing them to the one-health concept^9^. Effective forest-based initiatives should be multiplicated to educate people about the role of trees in urban human life^90^. Furthermore, an interplay between scientific approaches and local spirituality has been recently advocated in order to reach a more complete people awareness on nature elements and to enable more effective sustainable development and climate change adaptation^98^.

## Conclusions

In this time of climate crisis, tree urban plantation initiatives are multiplying in cities around the world as an attempt at human adaptation to new environmental conditions. While great interest in the scientific community has been devoted to ES survey methods, data elaboration and modelling, less attention has been given to the correct and effective communication and dissemination of the results.

Aula Verde is an innovation in the field of urban forestry because it combines ecology and ecosystem services with human health, social and artistic visions. The dialogue among different fields of expertise is often hampered by the use of different visions and vocabulary, and the challenge is to communicate within a common project. Even in the case of Aula Verde, although the statement was clear from the beginning, it was a challenge to merge the artistic, philosophical and scientific languages in a more academic description. The realisation of the first Aula Verde Aniene represents concrete experience, a living key study, to test and experience the transdisciplinarity and the interspecies design. This research confirms the statement of Aula Verde, which is at the same time an artwork (land art, more-than-human art piece, science-informed art piece, social sculpture), a NBS (realised with co-creation and active participation), a common place accessible to all, a forest medicine with emotional well-being. Looking for a shorter definition, we could say that Aula Verde is a Nature-Based Artwork. This definition tries to condense the many aspects of the Aula Verde project into a short sentence, but it seems incomplete. A common challenge is how to succinctly convey transdisciplinarity without giving up complexity. The picture 2 tries to give a conceptual visualisation of the definition of Aula Verde. A second challenge is to spread correct scientific information to citizens. The realisation of Aula Verde Aniene represents a pilot project aimed at opening a follow-up of similar actions, creating proximity between trees and communities. The creation of a multidisciplinary educational path has been an effective action for improving citizens’ awareness and quality of life. In this case, education was aimed towards a “knowledge-based society”^99^. Aula Verde is a project to reconnect inhabitants with ecosystems and is a common space made to take care of the ecosystems and all the living organisms that are part of it^7^.

Policy recommendations can be drawn from the study. Firstly, this project has demonstrated that a solid scientific foundation yields favourable outcomes when planning NBS and disseminating associated scientific concepts. This result can be addressed to local authorities and to international bodies financing NBS implementation. Involving research centres and universities in NBS management can aid in facilitating the effective implementation of these strategies.

Furthermore, NBS projects should be inspired by an artistic vision, responding to needs beyond functionality. To achieve this, NBS planning, management, and monitoring should involve architects, artists, and environmental scientists working together.

Finally, art can be considered as a best practice to bring citizens closer to natural ecosystems and build environmental awareness. The practice of art stimulates curiosity and emotions and, in certain contests, can be more efficient in conveying scientific ideas than using words or numbers.

During the “greenwashing era”, we receive a huge amount of information on the ecological value of trees, but communication about it is often characterised by oversimplification. In the described project, art and science interacted to transmit information to citizens, describing life and its complexities.

### Supplementary Information

Supplementary Information.

Supplementary Video 1.

## Data Availability

All data generated and analysed during this study are included in this published article (Supplementary Material files: SM 4, SM 6, SM 7, SM 8, SM 9, SM 10).
