# Supplementary material for: Aula Verde (tree room) as a link between art and science to raise public awareness of nature-based solutions
Source: Sci Rep. 2024 Feb 6;14:2368. doi: 10.1038/s41598-024-51611-9 (PMC10847515; doi:10.1038/s41598-024-51611-9)
Supplement: Supplementary file 1 — Supplementary Information. [file 41598_2024_51611_MOESM1_ESM.docx]

Supplementary material SM 1. Local dataset on Photosynthetically Active Radiation (PAR), temperature and precipitation recorded during 2015 in the meteorological station of the Urbe Airport (Rome).


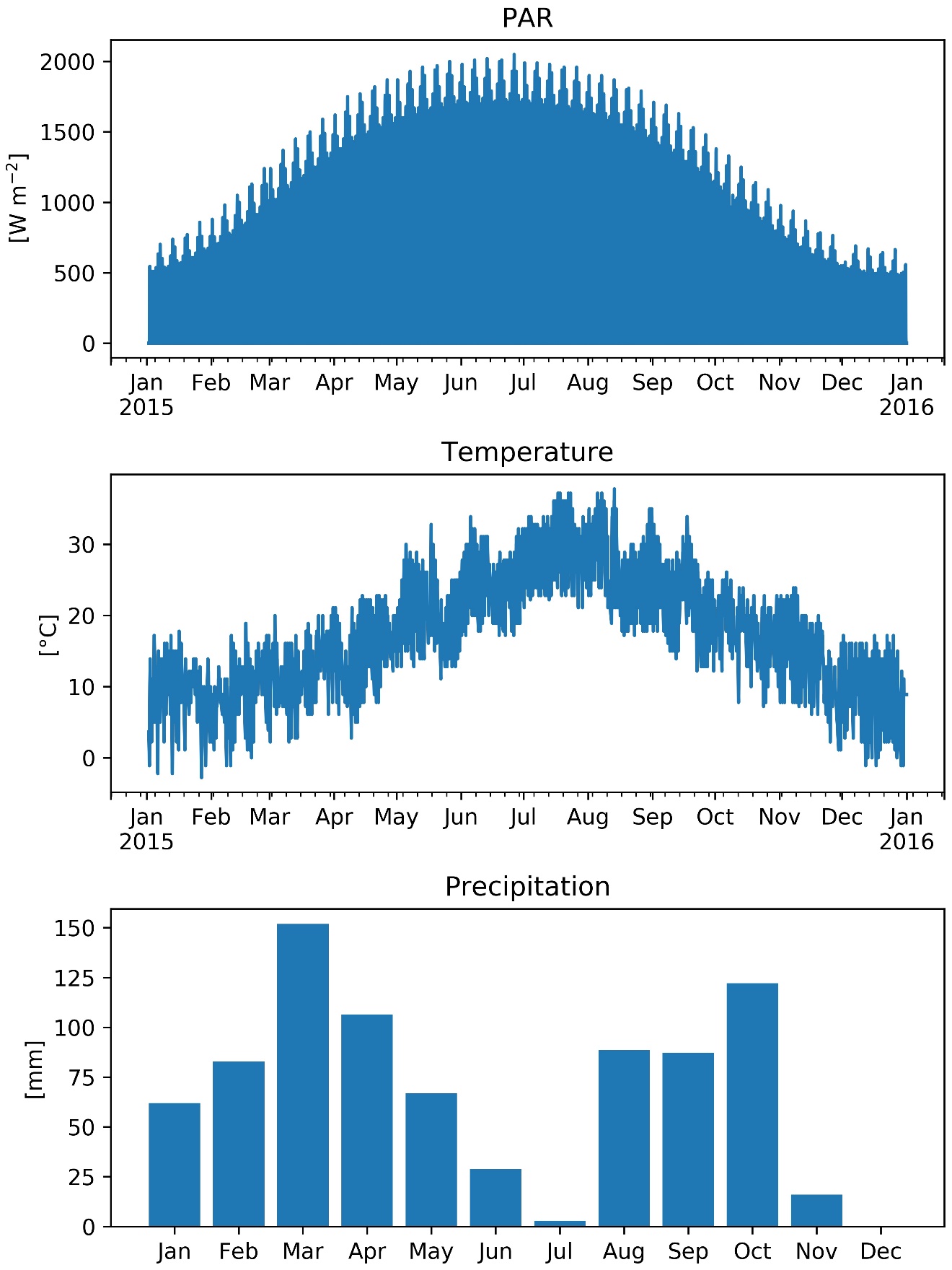


Supplementary material SM 2. Air quality data collected during 2015 in the meteorological station of the Urbe Airport (Rome). Concentration of Carbon Monoxide (CO), Sulfur Dioxide (SO_2_), Ozone (O_3_), Nitrogen Dioxide (NO_2_) and Particulate Matter (PM10 and PM25).


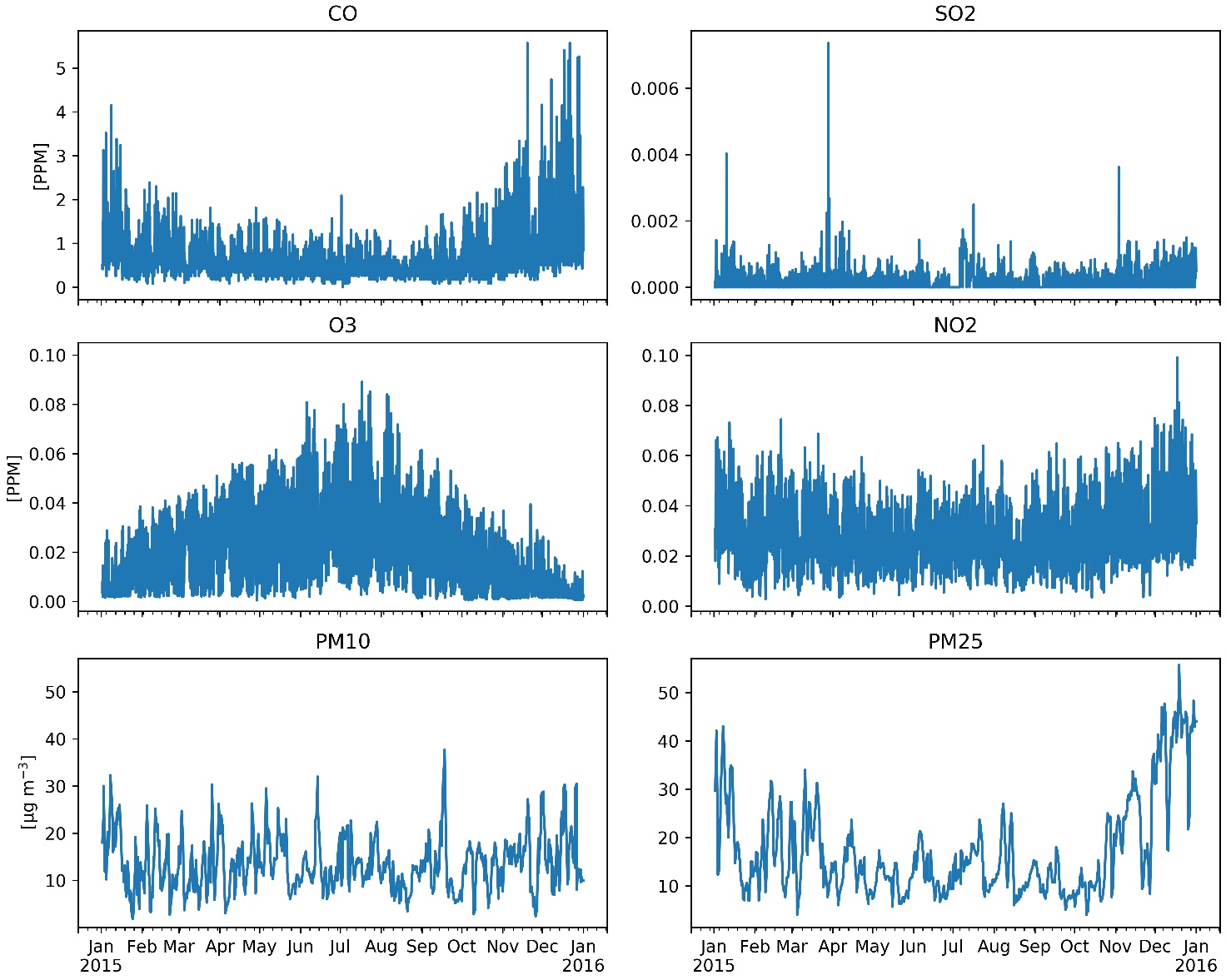


Supplementary material SM 3. Translation of the questionnaire text circulated among the contacts of local associations of Valle dell'Aniene park.

**Intro to the survey- “**We are a group of researchers, artists and cultural associations, and we need your help to assess the well-being that can result from the presence of greenery in the city.

The Aula Verde is located in the Aniene Park and is formed by concentric circles of trees that delimit a space dedicated to meeting and environmental education. The Aula Verde was conceived by artist Andreco and realized in April 2021 with the support of researchers from the CNR (National Research Council) and the active participation of citizens.”

**Questions** 1. How old are you?

0-18; 19-25; 26-35; 36-45; 46-55; 56-65; over 65.

2. What educational qualification do you have?

Middle school diploma, High school diploma, Degree, Master’s degree, PhD.

3. What benefits does the presence of trees provide? (optional)

Free answer.

4. Are you familiar with the Aula Verde in the Aniene Valley Park?

Yes; No.

5. How do you feel in the middle of Aula Verde (circle of trees)?

Uncomfortable, intimidated; Indifferent; Well, at ease.

6. What do you feel when you are in the middle of Aula Verde (circle of trees)?

Recreation: any, medium, a lot.

Aesthetic values: any, medium, a lot.

Spiritual values: any, medium, a lot.

Inspiration: any, medium, a lot.

Social relations: any, medium, a lot.

Physical health: any, medium, a lot.

Mental health: any, medium, a lot.

7. Do you think the Aula Verde helps raise awareness and respect for the ecosystem?

Yes; No.

8. Do you actively participate in environmental activities or associations?

Yes; No.

9. Would you like to participate in environmental activities or associations in the future?

Yes; No.

Supplementary material SM 4. Responses to the question 3 of the survey “What benefits does the presence of trees provide?”. This was an open-ended and optional question. The varied responses have been translated from Italian and classified in four categories of ecosystem services: cultural, regulating, supporting and provisioning.

Cultural ES: mental well-being (8 responses), moral well-being, well-being (7 responses), spiritual benefit (3 responses), psychological benefit (4 responses), mental regeneration, reconnection to the Naure (3 responses), nature contemplation, mark the flow of the seasons, a more open heart, solace, peacefulness (5 responses), source of imagination, emotional balance, landscape improvement (4 responses), aesthetic improvement (2 responses), relax (5 responses), stress relief (2 responses), please the eyes (2 responses), leads to listen, connects peoples, social aggregation (2 responses), social improvement, peace (3 responses), sense of beauty (9 responses), protection, source of harmony, give a meaning to the days, fun, delight, restoring green, the fragrances and the sounds similar to those in D’Annunzio's poems, freedom, healthy conversations,

Regulating ES: clean air (29 responses), cool shade (24 responses), mitigation of the effects of climate change (2 responses), rehabilitation, prevention of soil erosion (2 responses), soil remediation (2 responses), carbon dioxide uptake (3 responses), urban noise mitigation, climate improvement (2 responses), landslides prevention, pollution mitigation (3 responses), flood protection, calm,

Supporting ES: breath, make hearth breathing, oxygen (18 responses), habitats for animals (4 responses), refuge for animals (2 responses), habitat for birds (3 responses), refuge for insects, ecosystem preservation (2 responses), benefits for the environment (3 responses), functional soil, habitat for animal and vegetal species, nature conservation, flowers for bees.

Provisioning ES: fruits (3 responses), berries, renewable energy

Not classified responses: Life (7 responses), physical regeneration, a lot of environmental benefits, physical well-being (3 responses), a lot of benefits (3 responses), material benefits, care of the land, an opportunity for the environment, countless benefits

Supplementary material SM 5. Pictures showing the planting of Aula Verde and the data collection on the trees. Photo author: Futura Tittaferrante.


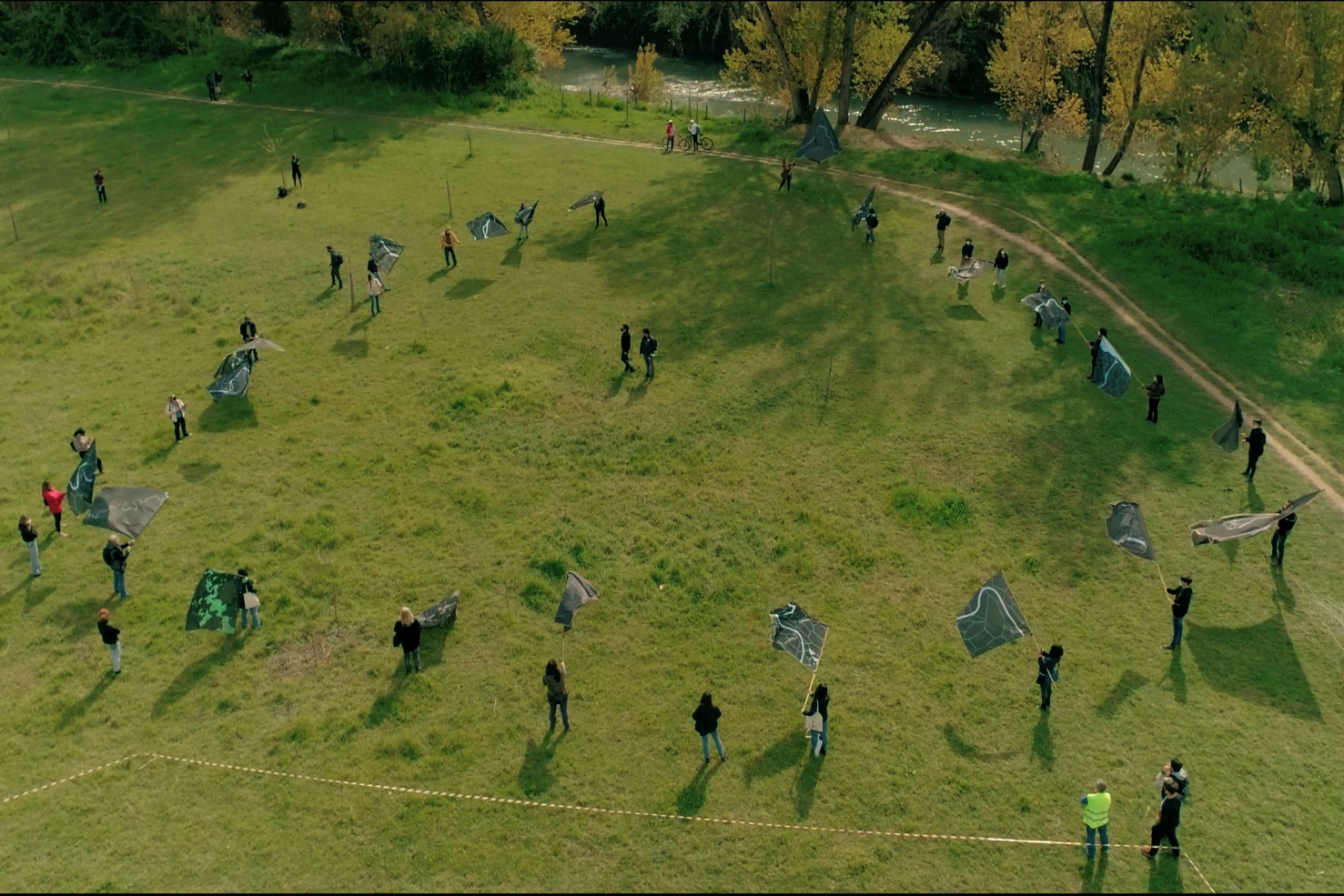

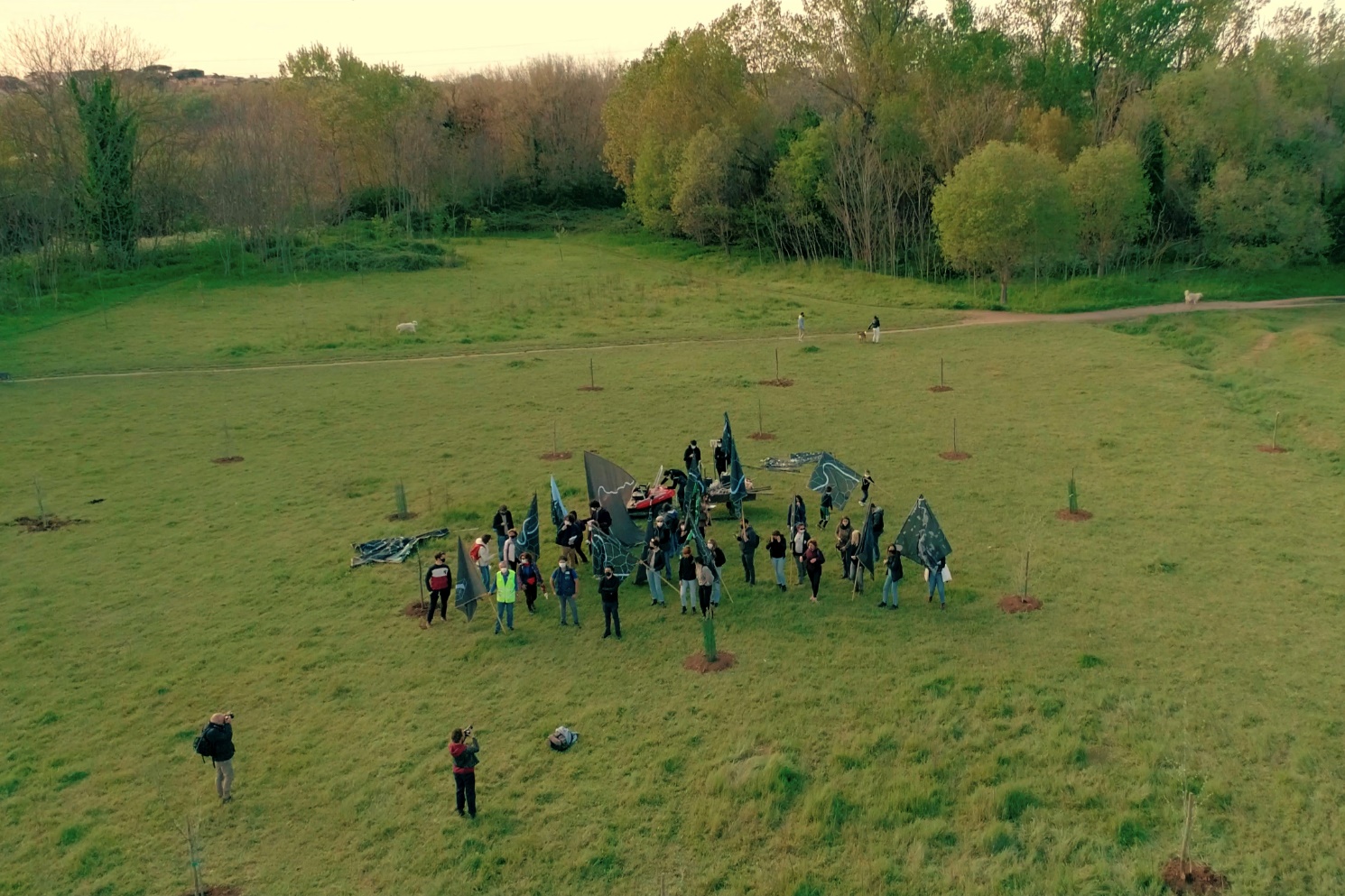


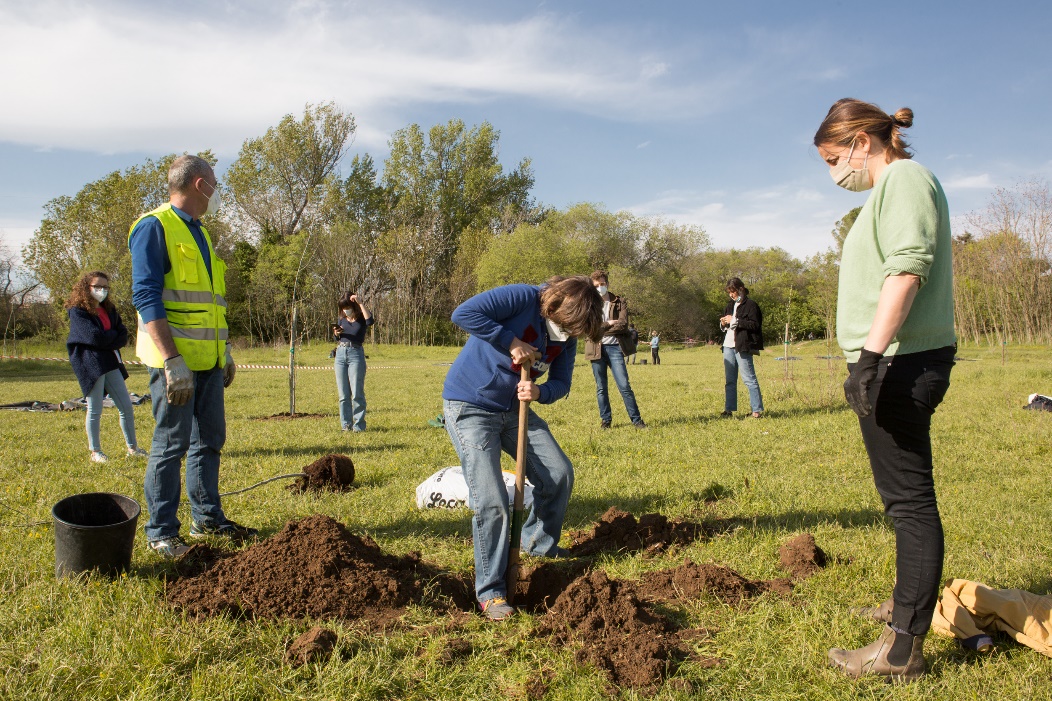

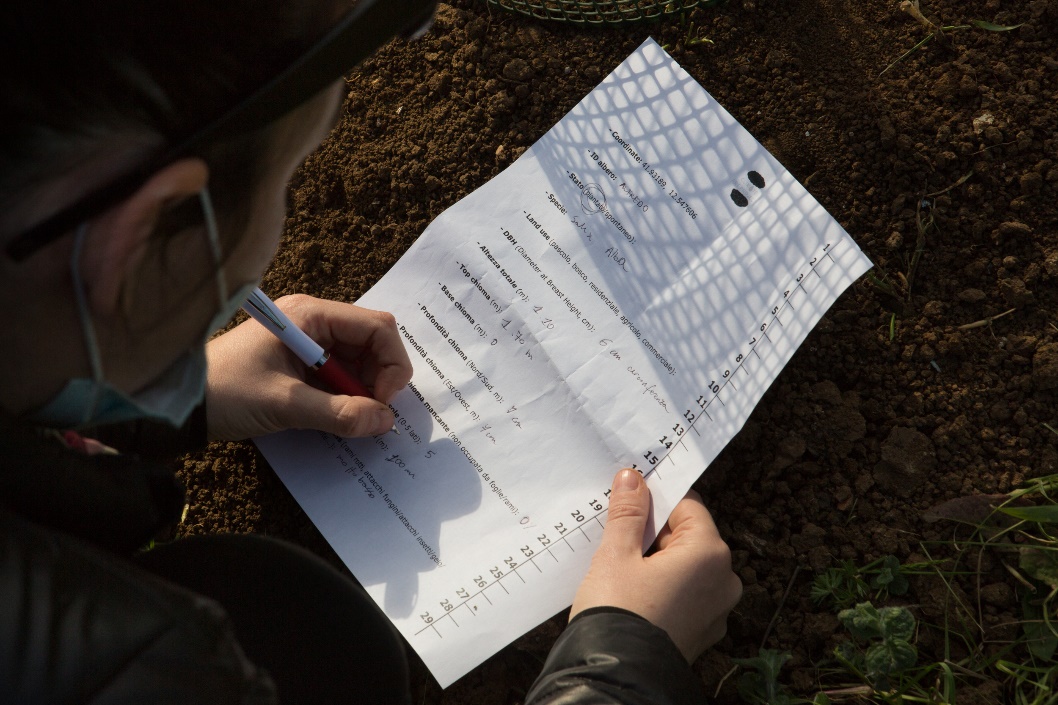

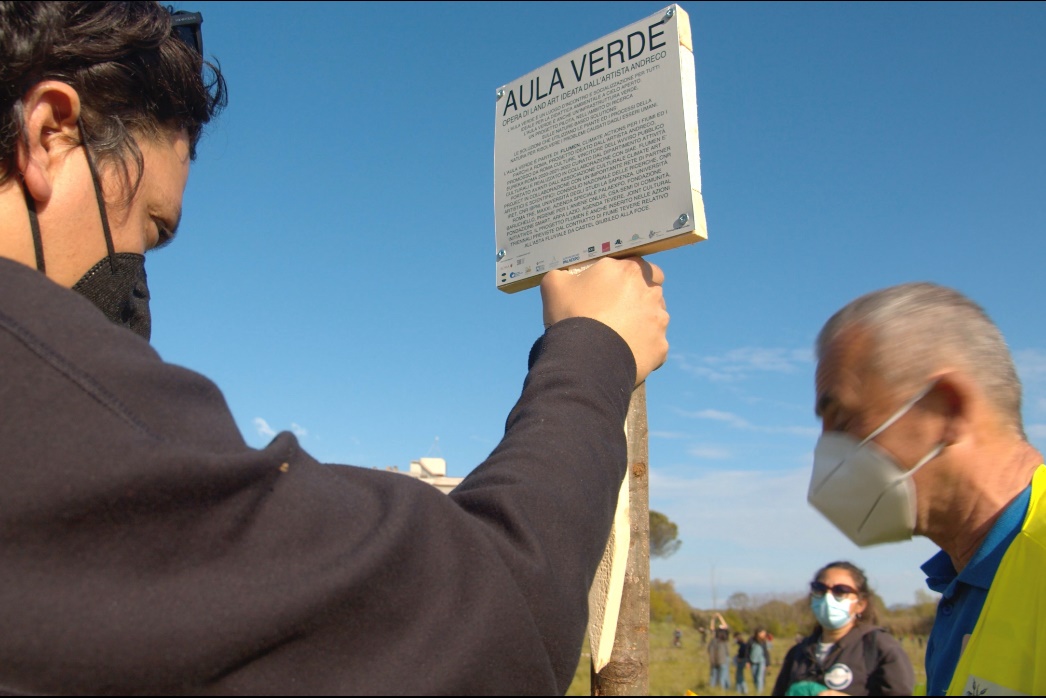


Supplementary material SM6. Row data from the questionnaire responses collected using Google Forms. The answers to question n. 3 have been translated in Supplementary Material SM 3

Supplementary Material SM7. Row data from i-tree model to assess the Carbon storage and sequestration (metric tons) of Aula Verde

Supplementary Material SM8. Row data from i-tree model to assess the transpiration and avoided run-off (m^3^/year) of Aula Verde at year 10, 20, 30 40 and 50 after planting

Supplementary Material SM9. Row data from i-tree model to assess the pollution removal (Kg of O_3_, Kg of NO_2_, Kg of PM10, Kg of PM25, Kg of CO, Kg of SO_2_) of Aula Verde from year 1 to year 50 after planting

Supplementary Material SM10. Row data from i-tree model to assess the tree cover (m^2^), total leaf area (m^2^), total basal area (m^2^) of Aula Verde from year 1 to year 50 after planting.
